# Elucidating the Structural and Minimal Protective Epitope of the Serogroup X Meningococcal Capsular Polysaccharide

**DOI:** 10.3389/fmolb.2021.745360

**Published:** 2021-10-14

**Authors:** Gian Pietro Pietri, Marta Tontini, Barbara Brogioni, Davide Oldrini, Stefania Robakiewicz, Pedro Henriques, Ilaria Calloni, Vera Abramova, Laura Santini, Suzana Malić, Karmela Miklić, Berislav Lisnic, Sara Bertuzzi, Luca Unione, Evita Balducci, Jérôme de Ruyck, Maria Rosaria Romano, Jesus Jimenez-Barbero, Julie Bouckaert, Stipan Jonjic, Tihana Lenac Rovis, Roberto Adamo

**Affiliations:** ^1^ Center for Proteomics, Faculty of Medicine, University of Rijeka, Rijeka, Croatia; ^2^ GSK Vaccines, Siena, Italy; ^3^ Unité de Glycobiologie Structurale et Fonctionnelle, Université de Lille, Villeneuve D’Ascq, France; ^4^ Chemical Glycobiology Lab CIC BioGUNE Technology Park, Derio, Spain; ^5^ Ikerbasque, Basque Foundation for Science, Bilbao, Spain; ^6^ Department of Organic Chemistry II, University of the Basque Country, Universidad Del País Vasco/Euskal Herriko Unibertsitatea, Leioa, Spain

**Keywords:** structural glycobiology, glycoconjugates, vaccines, *Neisseria* meningitidis, capsular polysaccharide

## Abstract

Despite the considerable progress toward the eradication of meningococcal disease with the introduction of glycoconjugate vaccines, previously unremarkable serogroup X has emerged in recent years, recording several outbreaks throughout the African continent. Different serogroup X polysaccharide-based vaccines have been tested in preclinical trials, establishing the principles for further improvement. To elucidate the antigenic determinants of the MenX capsular polysaccharide, we generated a monoclonal antibody, and its bactericidal nature was confirmed using the rabbit serum bactericidal assay. The antibody was tested by the inhibition enzyme-linked immunosorbent assay and surface plasmon resonance against a set of oligosaccharide fragments of different lengths. The epitope was shown to be contained within five to six α-(1–4) phosphodiester mannosamine repeating units. The molecular interactions between the protective monoclonal antibody and the MenX capsular polysaccharide fragment were further detailed at the atomic level by saturation transfer difference nuclear magnetic resonance (NMR) spectroscopy. The NMR results were used for validation of the *in silico* docking analysis between the X-ray crystal structure of the antibody (Fab fragment) and the modeled hexamer oligosaccharide. The antibody recognizes the MenX fragment by binding all six repeating units of the oligosaccharide *via* hydrogen bonding, salt bridges, and hydrophobic interactions. *In vivo* studies demonstrated that conjugates containing five to six repeating units can produce high functional antibody levels. These results provide an insight into the molecular basis of MenX vaccine-induced protection and highlight the requirements for the epitope-based vaccine design.

## Introduction

*Neisseria meningitidis* (Men) is a Gram-negative encapsulated diplococcus, capable of producing meningitis and sepsis in humans (Morelli et al., 2014; Fiebig et al., 2016; Ji et al., 2017). In recent times, thousands of cases and scores of deaths have been recorded around the globe. However, the sub-Saharan African meningitis belt is by far the most affected area in the latest years (Micoli et al., 2013; Morelli et al., 2014; Muindi et al., 2014; Lee et al., 2015; Chen et al., 2018; Oldrini et al., 2018).

Most pathogenic Men are coated by a capsular polysaccharide (CPS) (Pizza and Rappuoli, 2015) as it improves colonization through evasion of the host’s immune system (Fiebig et al., 2014) and allows survival in the blood of the host (Swain et al., 2017). Based on the chemical composition of the CPS, Men is subclassified into 12 serogroups, with A, B, C, W, Y, and X being the most clinically relevant ones (Micoli et al., 2013; Harale et al., 2015; Ming et al., 2018). Men CPS itself is highly immunogenic and elicits bactericidal antibodies in adult population (Fiebig et al., 2014); consequently, it has been widely used for the development of polysaccharide vaccines (Fiebig et al., 2014; Fallarini et al., 2015; Ji et al., 2017; Ming et al., 2018). More recently, Men CPS has been covalently linked to immunogenic protein carriers, such as the chemically detofixied diphtheria or Tetanus Toxins (DT and TT, respectively) and the nontoxic mutant of the diphtheria toxin, Cross-Reacting Material 197 (CRM_197_), to form glycoconjugates (Morelli et al., 2014). Men glycoconjugate-based vaccines, such as Menactra, Menveo, Nimenrix, and more recently MenQuadfi (targeting MenA, C, Y, W) (Berti et al., 2021), have overcome most of the limitations of using plain Men CPS, that is, the lack of memory response, IgM-to-IgG maturation, and ineffectiveness in children below 2 years of age (Muindi et al., 2014; Fallarini et al., 2015; Harale et al., 2015; Pecetta et al., 2016; Hlozek et al., 2018). Over the past years, a MenA-TT conjugate, MenAfriVac, has been introduced in the so-called meningitis belt, leading to almost eradication of the disease (Trotter et al., 2017).

Due to structural similarities with glycans from fetal gangliosides (Finne et al., 1983), this approach has been unfeasible for MenB, against which protein vaccines have been developed (Masignani et al., 2019).

MenX strains were first described in 1966 by Boris et al. (Garrido et al., 2012; Tanir et al., 2018), yet until recently, their association with invasive disease was not on pair with the other disease-causing serogroups (Ji et al., 2017). However, in the past years, several MenX outbreaks have been registered in the meningitis belt (Muindi et al., 2014; Chen et al., 2018; Ming et al., 2018). The surge of MenX has alerted the World Health Organization (WHO), reclassifying this serogroup as a major threat (Morelli et al., 2014). Particularly, after the introduction of MenA mass immunization in Africa, the increasing incidence of MenX infections has been attributed to MenA serogroup displacement (Morelli et al., 2014). Alternatively (Tanir et al., 2018), recent work from Ji et al. showed that a MenX strain, isolated from a bacteremia case in China, derived from a MenA strain due to a capsule switching event (Ji et al., 2017).

Considering the potential emergence of MenX-related meningococcal disease (Morelli et al., 2014; Ji et al., 2017), it is indisputable that MenX disease poses a threat to global health, making the development of a vaccine a top priority (Ji et al., 2017).

Several MenX vaccines are already in preclinical trials using MenX PS as a vaccine antigen as the leading strategy (Micoli et al., 2013). For example, a vaccine containing MenX CPS fragments conjugated to CRM_197_ has been successful at the preclinical stage (Morelli et al., 2014). A classic polysaccharide-protein conjugate approach is under investigation by the Indian Serum Institute for the development of a pentavalent *Men*ACXYW vaccine (NmCV-5). Other modern strategies include the vaccines containing enzymatic and chemically produced MenX oligosaccharides (OSs) (Micoli et al., 2013; Fiebig et al., 2016).

The MenX CPS is composed of a repeating unit (RU) of N-acetylglucosamine-4-phosphate residues held together by α-(1–4) phosphodiester bonds (Micoli et al., 2013). While this RU structure was first confirmed by ^13^C NMR in 1974 (Smith, 1974; Garrido et al., 2012), little is known about the minimal antigenic determinant of the polysaccharide. *In vivo* studies performed by Morelli et al. found that three RUs were the minimal antigenic portion of the CPS capable of eliciting protective antibodies (Morelli et al., 2014). A synthetic fragment of four RUs has also been tested *in vitro* (Harale et al., 2015); however, these short lengths are considered suboptimal to elicit a robust immune response compared to the polysaccharide conjugate. Therefore, although dynamic simulation studies have hypothesized that four RUs could be the minimal epitope required for eliciting an immune response (Hlozek et al., 2018), it is a general belief that longer fragments might be required to mimic the *in vivo* response achieved with conjugates of the native CPS (Morelli et al., 2014; Harale et al., 2015; Oldrini et al., 2018). In this context, enzyme-based or combined chemo-enzymatic approaches have been used at the preclinical level to develop conjugate vaccines based on oligomers of around 11 RUs which induced high levels of functional antibodies (Fiebig et al., 2016).

Despite these studies to investigate the potential of the MenX polysaccharide in vaccine design, both the structural antigenic determinant and minimal immunogenic epitope of MenX CPS have not been elucidated (Harale et al., 2015; Carboni et al., 2019). This minimal epitope is crucial to guide vaccine design particularly from synthetic approaches, where its length should ideally be short enough for practical synthesis while keeping representation of the native CPS conformation (Hlozek et al., 2018; Carboni et al., 2019; Oldrini et al., 2020a). Mapping interactions of glycans with protective antibody epitopes is becoming a powerful tool to select glycans for epitope-focused vaccines, eliciting long-lasting immunity and highly specific bactericidal antibodies (Soliman et al., 2020). This principle has been successfully applied at the preclinical level to generate glycoconjugate vaccines against *Clostridium difficile*, *S. pneumoniae*, Group B *Streptococcus*, and other bacteria (Broecker et al., 2016; Carboni et al., 2017; Schumann et al., 2018; Oldrini et al., 2020a).

Herein, we isolated the first bactericidal monoclonal antibody against the MenX polysaccharide, and through an integrated approach based on the enzyme-linked immunosorbent assay (ELISA), surface plasmon resonance, and saturation transfer difference nuclear magnetic resonance (STD-NMR), we characterized its affinity toward the CPS and the positions involved in binding. Fab was also crystallized to generate an *in silico* model for the recognition with MenX CPS. The information generated from epitope mapping was utilized for the preparation of conjugates from different oligomer lengths. Combined data on the antigenic determinant involved in mAb recognition and on the minimal immunogenic portion support the notion that the minimal structural and immunogenic epitope of MenX CPS is composed of five to six RUs.

## Results

### Selection and Immunochemical Characterization of a Functional Anti-MenX Murine mAb

**FIGURE 1:**
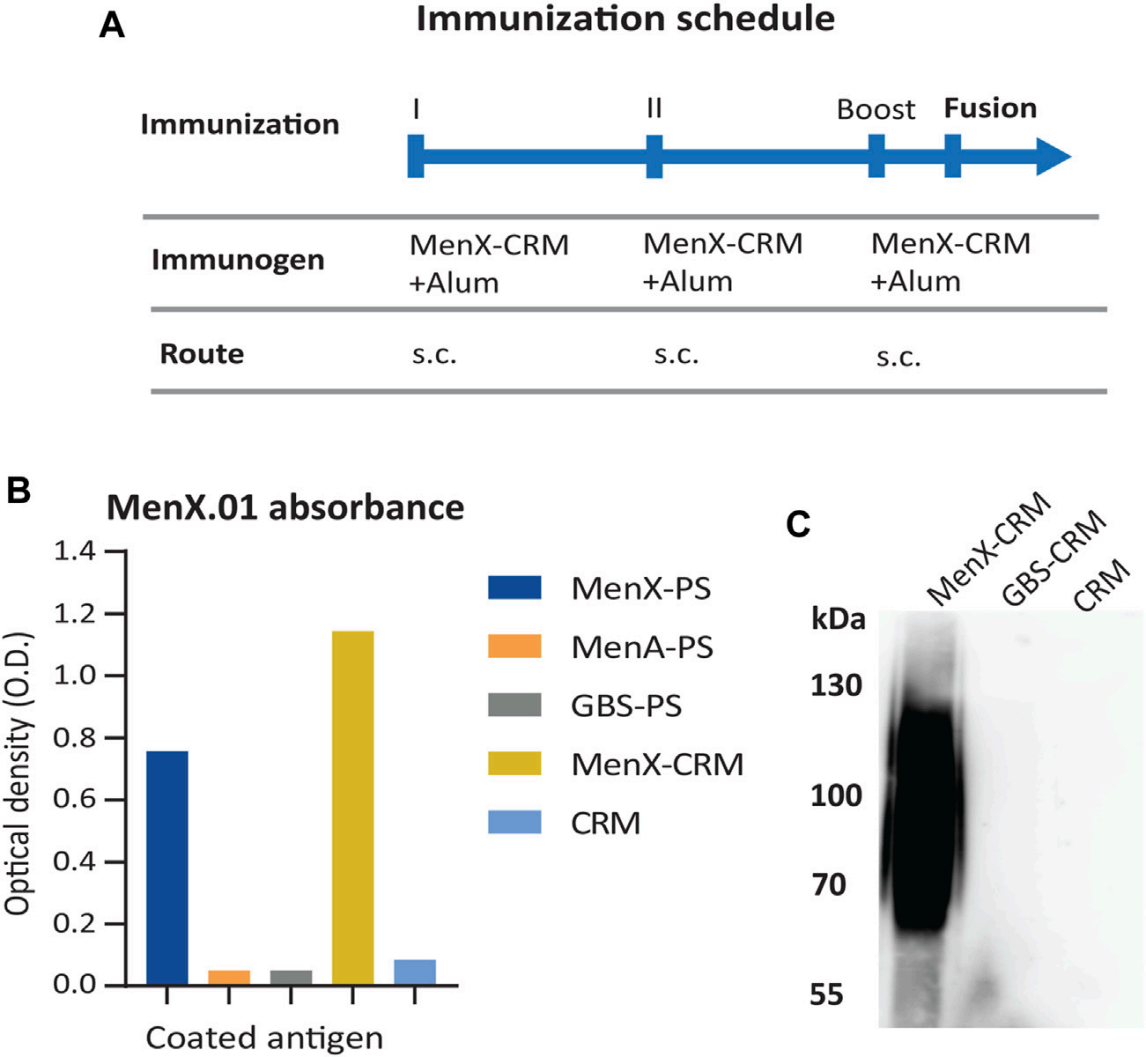
Specificity of the supernatants from the hybridoma cells producing mAb MenX.01 against the MenX polysaccharide. **(A)** MenX immunization protocol. **(B)** Cross-specificity test of purified MenX.01 mAb on ELISA plates coated with i) MenX-PS, ii) i) MenA-PS, iii) GBS-PSII, iv) MenX-CRM_197_ conjugate, and v) CRM_197_ protein. **(C)** MenX-CRM_197_ molecules were detected by western blot/imunoblot using MenX.01 mAb, followed by anti-mouse Fab-HRP. As a negative control, CRM_197_ and GBS PSII-CRM_197_ were used.

The anti-MenX CPS monoclonal antibody (mAb), clone MenX.01, was obtained using the hybridoma technology. The glycoconjugate of the *Neisseria meningitidis* serogroup X polysaccharide and the CRM_197_ carrier protein (MenX-CRM_197_) was used as an immunogen (Figure 1A). CRM_197_ was selected as it is present in the Menveo vaccine and has been shown to provide strong immunogenicity to MenX CPS (Micoli et al., 2013; Oldrini et al., 2018). Several attempts to immunize mice and obtain hybridoma cell lines were necessary to develop one monoclonal antibody that specifically recognizes the MenX polysaccharide. In total, close to 6,000 supernatants were tested for the binding assay on the MenX polysaccharide (MenX-CPS)-coated ELISA plates. Positive supernatants were retested, and in parallel, a cross-reactivity test was performed on an irrelevant meningococcal glycoconjugate. This resulted in a single hybridoma cell line that secreted an antibody specifically recognizing MenX-CPS, which was a kappa IgG1 isotype/subtype. Next, a large-scale mAb production and purification was performed, and the leading candidate, clone MenX.01, was purified from the serum-free medium by using one-step affinity purification in the milligram scale. The purified MenX.01 mAb was tested against several structurally different polysaccharides to confirm the lack of cross-reactivity between MenX CPS recognition and other bacterial carbohydrates (Figure 1B). The specificity of MenX.01 mAb was further confirmed by immunostaining of the MenX-CRM_197_ conjugate and CRM_197_ conjugated to group B *Streptococcus* GBSII as the control (Figure 1C), where the glycoconjugate MenX-CRM_197_, showed its typical band on SDS PAGE. The bactericidal activity of the new mAb MenX.01 was then assessed through the rabbit complement-mediated serum bactericidal assay (rSBA). Although a correlate of protection has not been estabilhsed yet for MenX, this assay is genreally considered a surrogate of protection for MenA, C, W, and Y vaccines (Acevedo et al., 2017), where it measures the vaccine-induced antibody potential to induce killing of Men in presence of the rabbit complement (Micoli et al., 2013). The bactericidal activity of the highly specific anti-MenX PS antibody, clone MenX.01, was tested *in vitro*. An rSBA titer of 1,024 at 0.98 μg/ml demonstrated the recognition of live bacteria MenX strain Z9516 and the capacity of triggering complement-dependent cytotoxicity by MenX.01 mAb.

### Conformational Analysis of the MenX Capsular Polysaccharide

MenX CPS is a homopolymer composed of α-(1–4)-phosphodiester-linked *N*-Acetyl glucosamines (Bundle et al., 1974). To understand if potential structural epitopes could be predicted, its conformational behavior and dynamic features were studied *in silico* using a combined theoretical [quantum mechanics (QM) and molecular dynamics (MD) calculations] and experimental (NMR) approach. Special attention was paid to the different torsion angles that define the glycosidic linkages and to the geometry of the six-membered rings.

**FIGURE 2:**
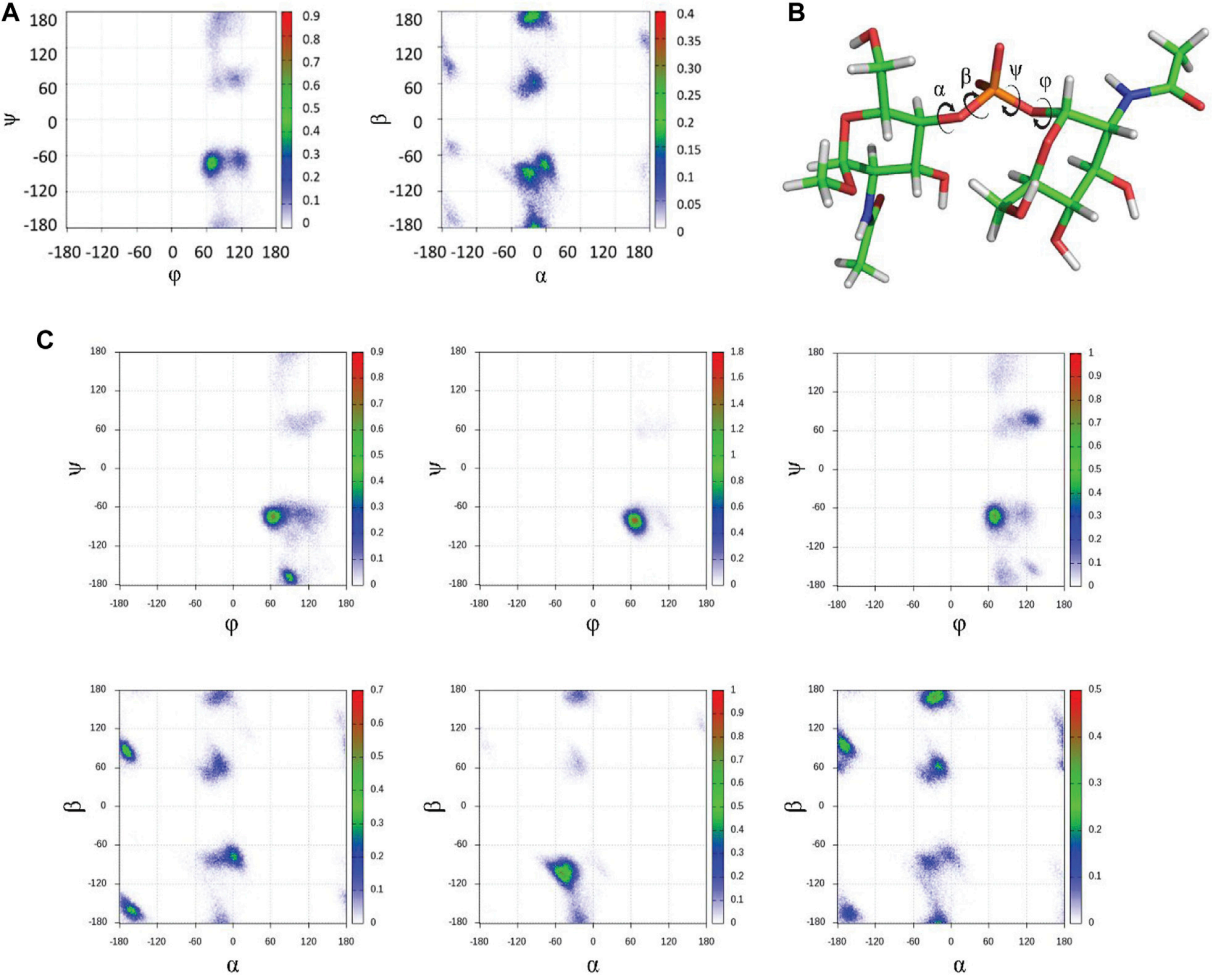
*In silico* conformational studies on MenX CPS. **(A)** Glycosydic linkage analysis for DP2. The φ/ψ and α/β plots from 1 μs MD simulations in explicit water are shown (GLYCAM06 force field) for MenX DP2. The conformational flexibility at β is evident, while the other three torsion angles display more restricted motion. **(B)** Structure of the global minimum for MenX DP2 as determined by MD calculations. **(C)** Selected φ/ψ and α/β plots for different contiguous disaccharide fragments of MenX DP12 from the 1μs MD simulations in explicit water (GLYCAM06) carried out for the dodecasaccharide.

First, to unravel the dynamic features at the glycosidic linkage of the MenX capsular polysaccharide while reducing the cost of the computational study, we performed a long (1.0 μs) MD simulation of the simpler disaccharide (DP2) repeating unit using the carbohydrate molecule-specific GLYCAM06 force field, explicit solvent molecules, and periodic boundary conditions as implemented in the Amber biomolecular simulation package (Salomon-Ferrer et al., 2013a). The results of the MD simulation indicated that in explicit water, MenX DP2 assumes a typical *exo-syn* conformation around the *ϕ* torsion angle, which is strongly stabilized by the *exo*-anomeric effect. The Ψ torsion angle largely populates the *syn-*conformation (ψ = -60°), which is favoured by steric effects, although minor excursions to other regions of the conformational map such as the *syn+* and *anti* conformations (+60° and 180°, respectively) are also possible. Instead, a higher degree of flexibility was observed for the α and β torsion angles. Specifically, the α angle shows a broad minimum around 0° (-60° ≤ α ≤ +20°), while β is characterized by a larger flexibility, with low energy minima at 180°, -60°, and 60° (Figure 2A). Taken altogether, the results from the MD simulation show that the energy profile for MenX DP2 explores different conformations, which differ for the combination of the flexible dihedral angles Ψ, α, and β, while keeping the *ϕ* torsion in the *exo*-anomeric conformation (Figure 2B).

The energy minima structures identified for the MenX DP2 disaccharide by the MD simulation were further evaluated using a QM approach at the B3LYP/6–31++g (d,p) level of theory using the Gaussian 09 suite of programs (Frisch GWT et al., 2016) to derive their expected NMR parameters that were compared to those experimentally determined. In particular, the analysis of the scalar (J) coupling constants was used to define the conformational distribution around the glycosidic linkage. The comparison between the experimental derived J-couplings and the calculated values confirmed the predominance of the *exo-syn* (*ϕ* = 60°) over the *exo-anti* (*ϕ* = -60°) conformation, which is probably present as a minor conformation (Supplementary Table S1, Supplemental Information). In agreement with the MD simulations previously described, the QM data also support the coexistence of different populations for the β torsion angle, while α is more restricted (−50° ≤ α ≤ 0°) (Supplementary Table S2, Supplemental Information).

Next, the identified structure of the DP2 disaccharide in its low-energy conformation was used to build a longer dodecasaccharide fragment (DP12) as a model of the entire polysaccharide. After submitting DP12 to 1.0 µs MD simulation, the results recapitulated those obtained for the simpler disaccharide with a few differences worth of mentioning. Briefly, the *exo-syn* conformation is preserved along the entire simulation. The ψ dihedral angle mainly populates the *syn-* (-60°) geometry, with minor excursions to the *syn+* (+60°) and *anti* (±180°) regions. A similar behavior for α and β dihedral angles is observed independently from the number of repeating units. Representative φ/ψ and α/β plots for DP12 are reported in Figure 2C. The analysis of the puckering of the six-membered rings showed that the low-energy ^4^C_1_ ring conformation is adopted by all residues along the entire simulation (data not shown). Overall, while a recent study has hypothesized that MenX CPS could display a large population of a helix-like geometry, especially for long polysaccharides, (Hlozek et al., 2018), the calculations performed herein for DP12 predict the existence of conformational flexibility mainly governed by the variability of β (mainly) and ψ to a minor extent.

### Selection of MenX CPS Fragments for Structural Studies

**FIGURE 3:**
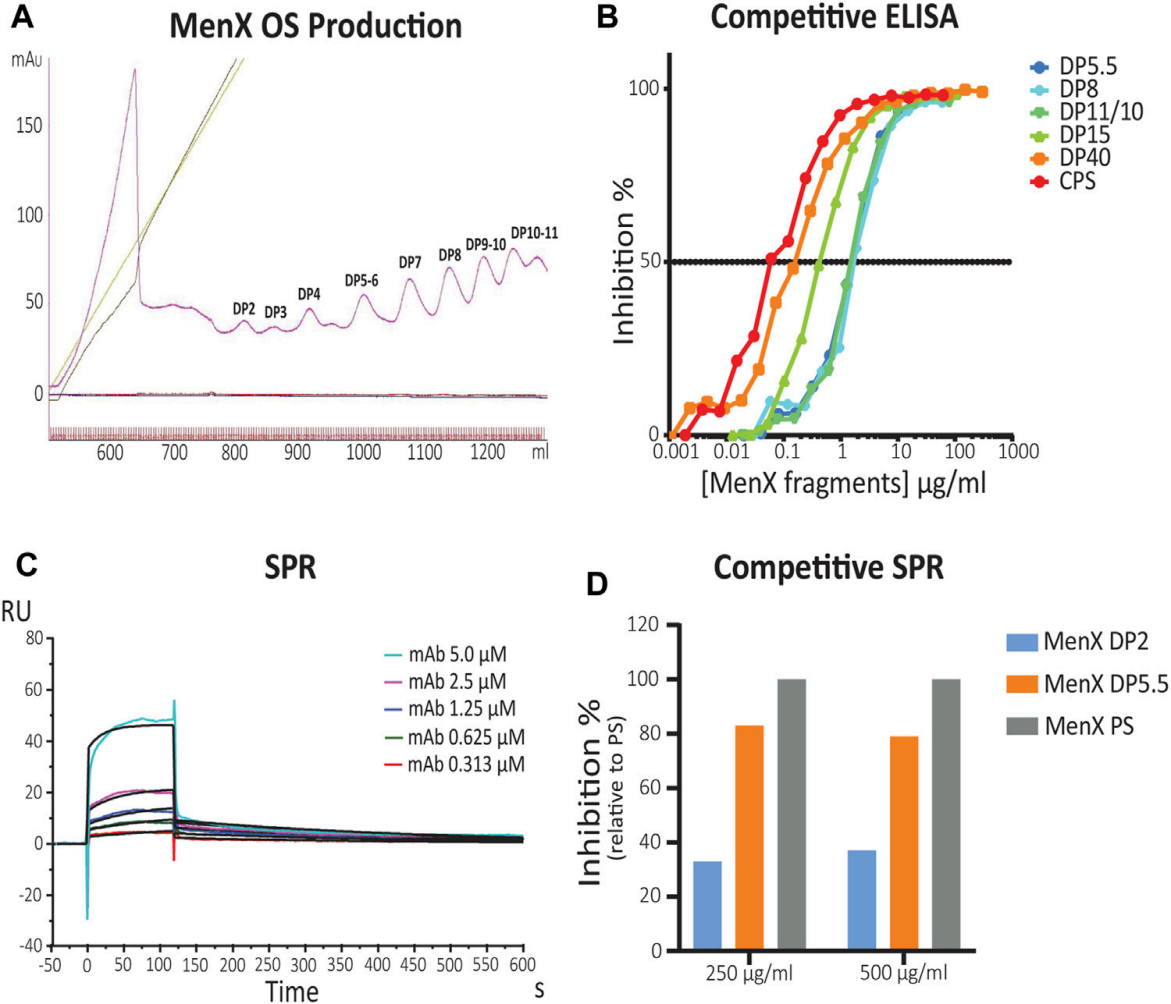
Identification of the MenX antigenic determinant by competitive ELISA and SPR. **(A)** Purification of different length MenX OS fragments. Sepharose Q column chromatography of depolymerized MenX CPS. **(B)** MenX.01 inhibition ELISA using different length inhibitors. Different MenX fragments were used as inhibitors, and MenX CPS and PFT (PBS containing 1% FCS and 0.05% Tween 20) buffer were used as the positive and negative controls, respectively. **(C)** SPR kinetic analysis of MenX.01. Binding kinetics and affinity constants of MenX.01 to MenX-CRM_197_ were determined by serial dilutions of the test antibody MenX.01. mAb was flown at a 0.3–5.0 µM concentration. Rmax was 9.92 ± 0.17 RU. **(D)** Comparison of MenX CPS, DP5.5, and DP2 relative capacity to block MenX.01 antibody binding by competitive SPR Study. Total capsular polysaccharide MenX DPS blocking was set at 100%.

The *in silico* analysis showed that MenX oligosaccharides display flexibility around the different torsional angles. Starting from this basic information provided by the calculations, the minimal MenX CPS portion able to recognize the functional MenX.01 mAb (Sharma et al., 2019) was empirically determined. We produced oligomers of different lengths, that is, average degree of polymerization (avDP) starting from the CPS. Mild acid hydrolysis and reaction monitoring by ^31^P NMR spectroscopy allowed to obtain the fragments (Supplementary Figure S1, 2). From the final sample with avDP 11.7, we purified oligosaccharide (OS) fragments in the DP range from 1 to 11 (Figure 3A).

Competitive ELISA was carried out using different concentrations of the generated OS fragments (DP5.5–40 range) as inhibitors. MenX.01 mAb was incubated with increasing amounts of different OS fragments and later transferred to MenX CPS immobilized on ELISA plates (Figure 3B). The absence of the primary antibody was used as a negative control. Inhibition of mAb at shorter lengths was comparable between DP5.5, DP8, and DP10.5. The inhibition was increased by 0.5 log with DP15 and by 1 log with the DP40 fragment, and the latter was slightly increased with the CPS. Therefore, competitive ELISA with the newly developed bactericidal MenX.01 mAb confirmed length-dependent recognition of the different fragments, that is, shorter fragments inhibited the interaction only at higher concentrations. The results also showed that avDP5.5 OS was sufficient to fully inhibit the binding of mAb to the native CPS, thus containing the minimal epitope. This is in line with previous reports showing that four RUs were not sufficient to inhibit the binding of rabbit anti MenX specific serum unless exposed as a protein conjugate (Harale et al., 2015).

To measure the binding kinetics of MenX.01 mAb and to examine its binding to shorter DP fragments, additional studies were performed using surface plasmon resonance (SPR). For this purpose, an avDP15 MenX conjugated to CRM_197_ was immobilized on a CM5 chip *via* the 1-Ethyl-3-(3-dimethylaminopropyl)carbodiimide (EDC) chemistry (pH 5) at a level of 458 RUs. This methodolgy was preferred to the immobilization of mAb because the negatively charged MenX polysaccharide flown onto it would be repsuled by the carboxylmethylated dextran matrix of the chip. The interaction was fit through the 1:1 Langmuir binding model. The equilibrium constant K_*d*_ (µM) of 0.32 ± 0.04 x 10^−6^ fitted based on the kinetic constants of k_*a*_ = 8.64 x 10^3^ M^−1^ s^−1^ and k_*d*_ = 2.75 × 10^−3^ s indicates a submicromolar affinity of the antibody for the MenX polysaccharide presented as a CRM_197_ conjugate (Figure 3C). A similar K_*d*_ (µM) of 0.54 ± 0.04 x 10^−6^ was measured flowing Fab. The difference with the K_*d*_ obtained with mAb was less than 2-fold and can be considered within the experimental variability. The SPR kinetic analysis of MenX.01 mAb showed that it binds with relatively fast on- and off-rates and moderate affinity to MenX, as typical for low-affinity carbohydrate–protein interactions. In a competitive SPR study, we confirmed that DP5.5 retains an almost complete (75%) capacity to block MenX.01 binding, compared to CPS (Figure 3D). On the other hand, the shorter fragment (DP2) had a very weak inhibitory capacity.

To gain further insights into the impact of carbohydrate length on binding, isothermal titration calorimetry (ITC) of OS in complex with MenX0.1 mAb was performed. The obtained data indicated that the affinity for MenX.01 mAb was very similar for DP5.5 and 7 (Supplementary Table S3 and Supplementary Figure S4, Supplemental Information), confirming that five to six RUs are sufficient to strongly bind to mAb. The interactions of the antibody with MenX fragments appeared largely entropically driven, with only a small enthalpic contribution. The affinity (K_*d*_), as measured by ITC, varied from ∼2 to 3 µM for the smaller DP5.5–7 to 0.80 µM for DP9 until ∼0.3 µM for avDP15 by SPR (Supplementary Table S3, Supplemental Information, and Figure 3C, respectively). The reduction of the K_*d*_, ranging from 2-fold for DP9 up to 10-fold for avDP15 could be ascribed to the increasing multimeric presentation of the epitope in longer chains.

### Mapping of the MenX Antigenic Determinant by STD-NMR

**FIGURE 4:**
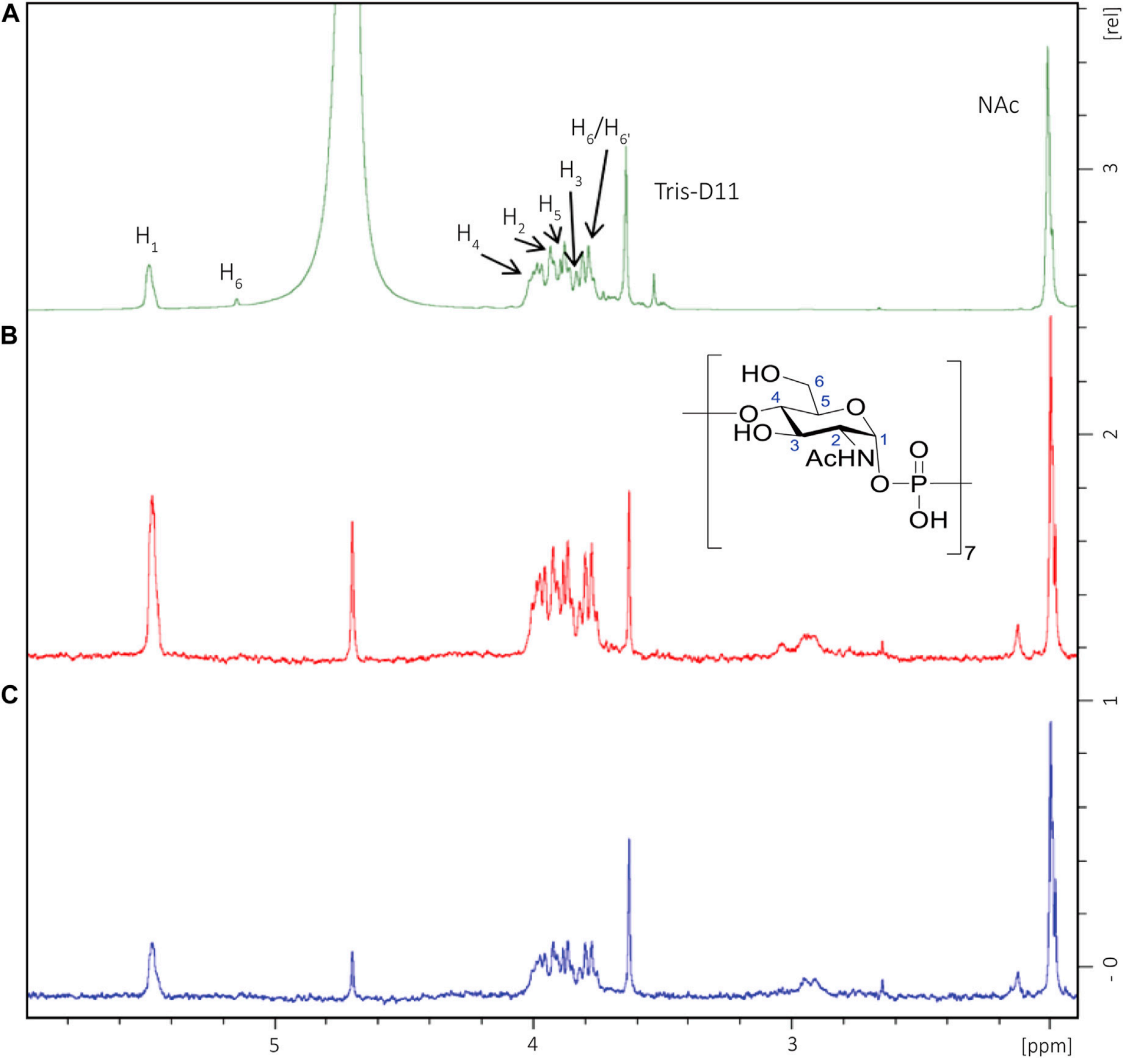
STD-NMR (D_2_O, 600 MHz) of a DP7 fragment in complex with MenX0.1 mAb. **(A)** ^1^H NMR of the oligosaccharide in the presence of MenX0.1 mAb (50:1 M ratio). **(B)** STD NMR spectrum obtained for the complex upon on-resonance irradiation at 7 ppm; **(C)** STD-NMR spectrum obtained for the complex upon on-resonance irradiation at 8 ppm. All sugar ring protons display transfer of saturation, with higher relative intensities for positions H-1 and H-4.

Considering that above a length of five to six RUs, the capacity to bind to antibodies was similar to the CPS, the interaction of DP7 and mAb MenX.01 was investigated by STD-NMR to map positions involved in binding. DP7 was selected as sufficiently long to contain the presumed epitope. The ^1^H NMR spectrum is characterized by a distribution of the ring protons within a small range of chemical shifts, which renders possible to make only qualitative considerations. Following irradiation of the DP7-mAb complex at 7 or 8 ppm, it clearly appeared that all the protons from MenX repeating units were receiving transfer of saturation (Figure 4), particularly the positions H-1 and H-4, which are held together by the phosphodiester bridge connecting the proximal units. This indicates that the area surrounding these charged groups is likely involved in strong interactions with the binding pocket. Since the hydrogen atoms of the GlcNAc residues in the typical ^4^C_1_ chair conformation point toward different spatial directions, the observed STD-NMR response is the result of the overall contribution of the different sugar units along the DP7 chain.

### MenX.01 Fab X-Ray Crystallography

To determine the exact binding epitope of the MenX.01 antibody on MenX sugar, we approached co-crystallization studies. For crystallization purposes, we produced different types of Fab fragments: Fab enzymatically obtained by digestion of the MenX.01 IgG1 antibody with papain and recombinant Fabs, with and without His tag, by production in transiently transfected HEK293T cells. All the three Fab fragments were successfully purified and functional (the enzymatic Fab and the recombinant His Fab shown in Supplementary Figure S3A–D), but none yielded crystals in co-crystallization studies. Then, we produced a fourth Fab - IgG2a recFab-His. Functionality and an affinity close to that of the original MenX.01 antibody were demonstrated for this Fab (Supplementary Figure S5, Supplemental Information). Co-crystallization studies of IgG2a recFab-His with the first smaller fragment of DP4 yielded crystals for X-ray analysis which diffracted at a resolution of 2.16 Å (Supplementary Figure S6, Supplemental Information). Unfortunately, the crystals contained Fab alone and not of the Fab-DP4 complex. Attempts to soak the crystals with millimolar concentrations of DP5.5 and DP4 were without success. Therefore, the obtained crystal was used for docking studies as confirmation of the NMR data.

### *In silico* Docking Studies of MenX OS Complexed With mAb

**FIGURE 5:**
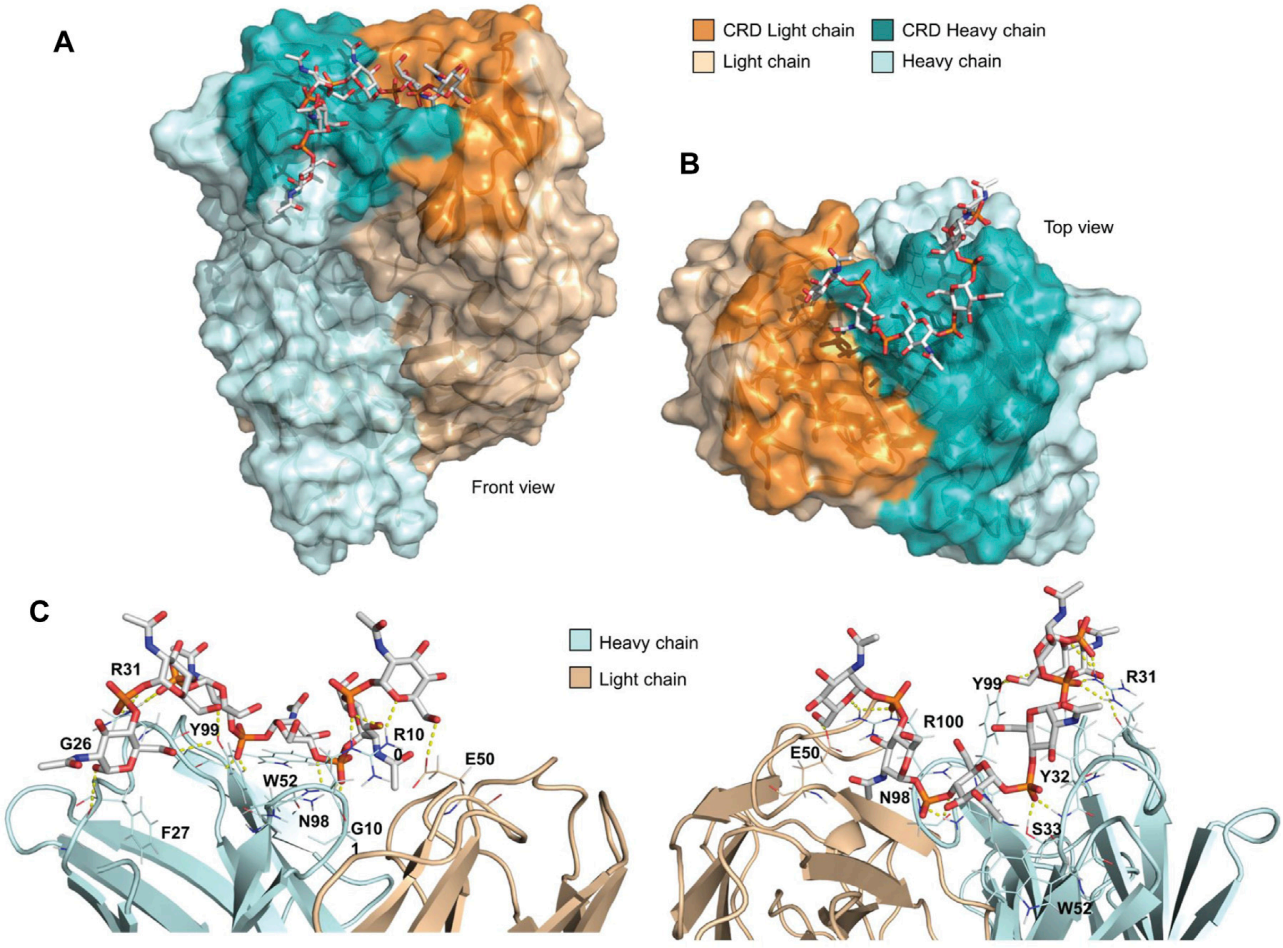
Docking of DP6 with MenX.01 Fab. **(A)** Top and **(B)** side view of the docking pose. DP6 engages the mAb binding pocket from the heavy to the light chains in the CRD. **(C)** Detailed intermolecular interactions in the docked-minimized structure of the complex between MenX.01 Fab and MenX DP6 OS. The amino acids that contribute to the binding are indicated.

To gain further insights into the molecular basis of recognition, the MenX hexasaccharide (DP6) was docked into the carbohydrate recognition domain (CRD) of the Fab region of MenX.01 mAb. The DP6 structure, corresponding to the central section of DP12 obtained by the MD simulations, was used as a representative of the DP5.5 MenX minimal epitope (Figure 5). The CRD of MenX.01 mAb shows an extended U-shaped groove running from the heavy to the light chains. Interestingly, most of the residues composing the CRD are positively charged amino acids (K5, R31, K75, K81, R97, and R100 in the heavy chain and K45 and R55 in the light chain), which confer a high positive charge to the surface (Supplementary Figure S7, Supplemental Information). Fittingly, the negatively charged phosphate groups of MenX DP6 may, at least partially, satisfy the positively charged surface. Thus, DP6 was docked into the CRD guided by the possible electrostatic intermolecular interactions among these groups. Next, a docking-minimization protocol of the complex was performed using the MAESTRO (Schrödinger) suite of programs (Schrödinger (2021). Maest, 2021). According to the calculations, the complex was conformationally stable and most of the intermolecular interactions were maintained, while new ones were found. In detail, R31 and R100 establish electrostatic interactions with the phosphodiester groups at the termini of the oligosaccharide chain. Additionally, all six residues of DP6 participate in hydrogen bond intermolecular interactions. In total, nine hydrogen bonds within residues S33, N98, Y99, R100, G101, G26, and E50 stabilize the complex, with four of them mediated by the phosphodiester groups all along the carbohydrate chain (Figure 5). Finally, nonpolar patches from the aromatic side chains of residues W52, Y99, Y32, and F27 also provide hydrophobic interactions to the DP6 oligosaccharide (Figure 5C). Interestingly, the phosphodiester groups at the edges of the DP6 oligosaccharide are instrumental to anchor the oligosaccharide chain to the mAb CRD through salt bridges with the R31 and R100 residues at the termini of the CRD. Additionally, the internal sugar residues establish a variety of intermolecular interactions all along the extended groove. This interaction pose is in agreement with the STD NMR outcome, which suggested that DP6 is globally involved in mAb recognition.

### Immunogenicity Studies

In order to study the impact of the saccharide chain length on the sugar immunogenicity and assess whether the 6-mer identified as a putative minimal epitope was able to elicit a robust and protective immune response, the DP5.5, 10, and 20 fragments were conjugated to CRM_197_ to be tested in a mouse animal model. DP10 and 20 were selected as comparators to DP5.5 in order to cover sufficiently different oligosaccharide sizes and taking into account that the strong immunogenicity of fragments longer than DP10 is well known (Micoli et al., 2013; Oldrini et al., 2018). Conjugation of MenX OS was achieved through a three-step procedure involving 1) reductive amination with a dihydrazine linker to insert a hydrazine moiety and 2) following reaction with di-*N*-hydroxysuccinimidyl adipate to transform the compound in a half ester for 3) final coupling to the protein carrier. The occurrence of conjugation was assessed by sodium dodecyl sulfate polyacrylamide gel electrophoresis (SDS-PAGE) and high-performance liquid chromatography (HPLC) (Supplementary Figure S8, Supplemental Information). The formed glycoconjugate was chararacterized by microBCA and anion-exchange chromatography with pulsed amperometric detection (HPAEC-PAD) analyses for quantification of the protein and unconjugated/conjugated carbohydrate components, respectively (Micoli et al., 2013; Oldrini et al., 2018). The prepared glycoconjugate vaccines were administered to mice at days 1, 14, and 28 using a 1 µg/saccharide of each biomolecule. Sera sampling was collected 14 days after the second and the third doses. Sera were analyzed for anti-MenX PS IgG content by ELISA and for antibody functionality by SBA.

**FIGURE 6:**
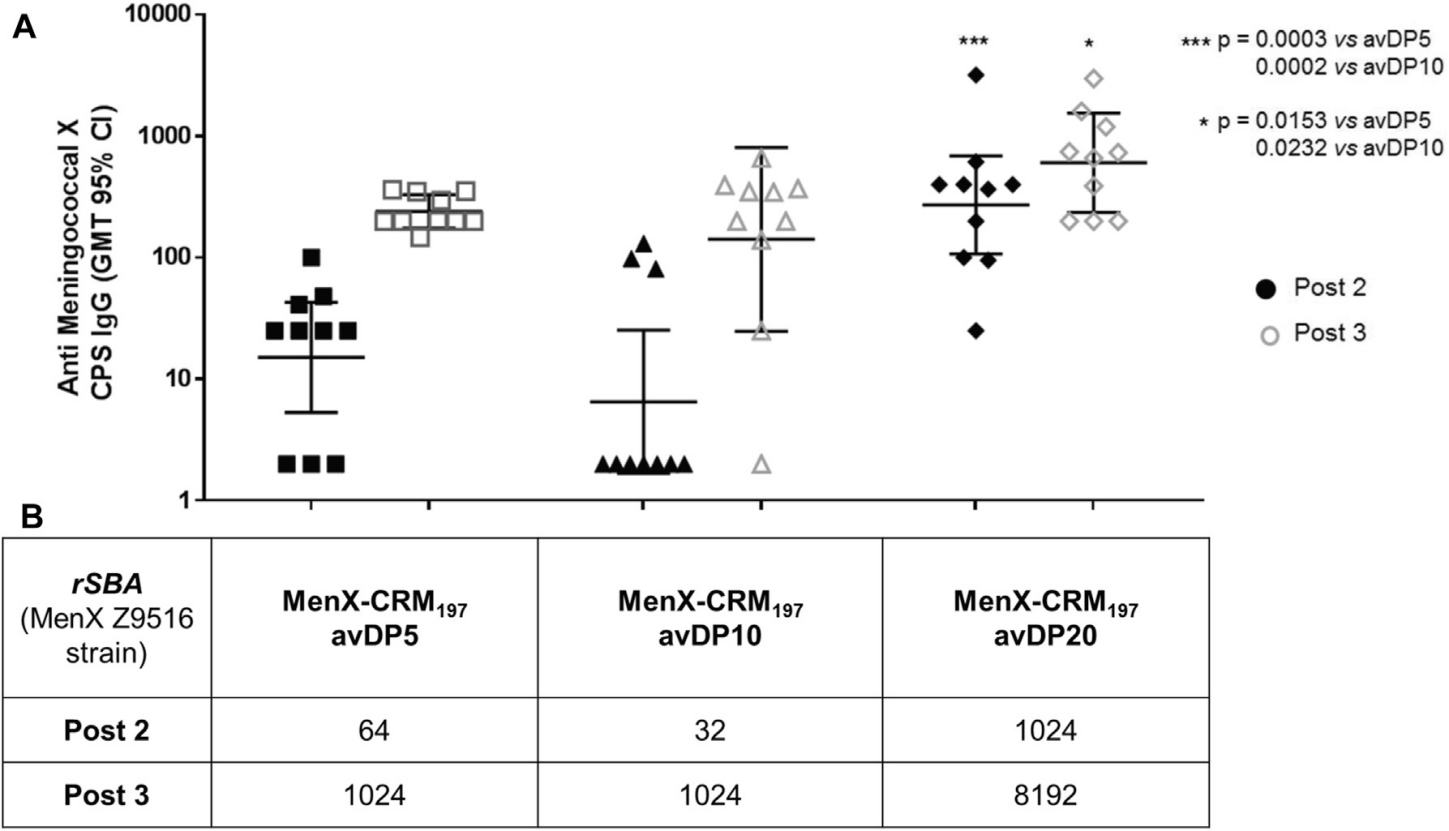
Immunogenicity of different length MenX OSs conjugated to CRM_197_. **(A)** ELISA geometric mean IgG titers and **(B)** SBA titers measured after the second and third doses are reported. Data are obtained from immunization of eight female BALB/c mice by subcutaneous injection of glycoconjugates at a 1 µg dose in saccharide content using alum phosphate as an adjuvant. Mice received the vaccines at days 1, 14, and 28. Mice were bled at days 0, 27, and 42. Dots represent single mice serum. Statistics was calculated with Mann–Whitney.

All the vaccines were able to induce a specific antibody response against the native MenX PS after the second dose that was boosted with the third dose (Figure 6). DP5.5 was able to induce IgG levels comparable to the conjugated DP10 and with similar functional activity, clearly indicating that this sugar length represents the minimal epitope capable of inducing a strong immune response. Of note, no treshold of protection is known for MenX CPS. The conjugated DP20 induced the best response from the set in terms of both antibodies and SBA titers, highlighting that further optimization of the immune response can be obtained by long fragments as a result of the multiple exposition of the minimal epitope along the polysaccharide chain. Previuos studies have shown no significant differences after the administration of conjugated avDP15-20 and avDP80-100, so we presume that avDP20 already mimics quite well the full CPS (Micoli et al., 2013).

## Discussion

In this study, we developed a highly specific antibody against the MenX polysaccharide, clone MenX.01, which was shown to be bactericidal. Our structural and immunogenicity data converged, establishing that the MenX minimal epitope is contained within five to six RUs (DP5.5).

To our knowledge, currently, there is only one monoclonal antibody, mAb 10B5F10, previously developed by Reyes et al. (2014), which recognizes MenX CPS, whose bactericidal activity, however, was not assessed. Our bactericidal mAb is an IgG1 subtype, which could be connected to the dominance of this subclass production induced by alum adjuvanted vaccination with glycoconjugates (Richter et al., 2004; Uddin et al., 2006).

MenX.01 mAb was able to induce bactericidal killing at a concentration as low as 1 μg/ml (rSBA of 1,024). An rSBA titer of ≥8 has been accepted as the correlate of protection for Men protective sera (Findlow et al., 2019); however, SBA protection threshold has not been defined for purified mAbs. Nevertheless, this protective mAb concentration appears realistic within the normal range of IgG in plasma since other immunization studies in mice using pneumococcal conjugates have produced specific anti-PS IgG antibody concentrations in sera of ∼10 μg/ml (Lee et al., 2003). Moreover, we found our rSBA titer similar to anti-MenA PS mAb 7E1F7, which reported rSBA titers in the range of 0.49 to 0.122 μg/ml (Madariaga et al., 2018). Altogether, this is evidence that the produced mAb is bactericidal at physiological IgG concentrations in sera.

In the case of homopolysaccharides, such as MenX CPS, identification of the conformation and orientation of the bound epitope to the corresponding antibody are challenging (Haji-Ghassemi et al., 2015). Therefore, the first step was to have an estimate of the shortest OS which contained the minimal antigenic determinant for further characterization. In our competitive ELISA study, the OS fragment avDP5.5 achieved full inhibition of the mAb-CPS binding. Furthermore, longer fragments, DP8 and DP10/11, did not increase the inhibition. This is an indication that the binding epitope is preserved above five RUs. Shorter OS fragments, such as DP4, could be considered suboptimal epitopes. The higher inhibition observed with DP15 and larger fragments is most probably due to a multivalency effect. These longer fragments support multiple binding to their repeated epitopes, while no specific conformational structure could be predicted *in silico* for MenX CPS, and SPR differences in the K_a_/K_d_ values from mAb and Fab highlighted that an avDP5.5 was able to bind to mAb similar to the CPS.

Of the range of Fab fragments that we successfully produced and purified, only recombinant Fab-IgG2a-His yielded crystals. Co-crystallization with sugar fragments proved to be unsuccessful (Haji-Ghassemi et al., 2015). *In silico* prediction on the MenX CPS conformation showed a high degree of flexibility in the polymer which prevents the formation of a preferential secondary structure, as opposed to a recent study highlighting potential formation of a helical structure for a length above six RUs (Fallarini et al., 2015). Docking studies with a six RU fragment and the crystal structure of Fab along with STD-NMR analysis showed that indeed this length is sufficient to fully occupy the binding pocket. A network on hydrogen bonds involving the charged phosphate groups along with additional nonpolar patches would play a relevant role in stabilizing such interactions.

Finally, the identified epitope recognized by the functional mAb was conjugated to a carrier protein demonstrating to elicit an immune response similar to a longer avDP10. This clearly indicates that a length of five to six RUs contains the minimal epitope of MenX CPS. The functional antibody levels were further increased for a conjugated avDP20 as a result of multimeric presentation of the identified epitope. Optimization of the presentation modality or different carrier proteins might be needed to further improve immunogenicity of the identified minimal epitope (Oldrini et al., 2020b).

In conclusion, our work identified a length of five to six RUs as a minimal structural and immunogenic epitope of the MenX capsular polysaccharide. Further effort will be devoted to unravel fine details of the recognition of functional antibodies. This study highlights the importance of a structural approach for the rational selection of the polysaccharide fragments for vaccine development. In addition, this study can guide the design of minimal epitope-based vaccines using synthetic or enzymatic methods.

## Materials and Methods

### Development of the anti-*Neisseria meningitidis* Serogroup X Polysaccharide (MenX-CPS) Monoclonal Antibody, Clone MenX.01

The glycoconjugate of the *Neisseria meningitidis* serogroup X polysaccharide with the CRM_197_ carrier protein (MenX-CRM_197_) (GSK, Siena, Italy) was used for BALB/c mice immunization (in groups of three mice). Immunogens were prepared by mixing the MenX-CRM_197_ stock [2 µg polysaccharide content, diluted in phosphate-buffered saline (PBS)], with Alhydrogel^®^ adjuvant 2% (aluminium content: 9–11 mg/ml) in a 1: 9 alhydrogel: MenX-CRM_197_ ratio. The immunogen was prepared on the day of immunization and gently mixed at room temperature (RT) for 4–5 h. Mice were subcutaneously immunized with the MenX-CRM_197_ conjugate and Alhydrogel^®^ adjuvant two times, at day 0 and 14. After the second immunization, the sera of immunized BALB/c mice were screened for antibody titers against the MenX polysacharide (MenX CPS) (GSK, Siena, Italy) by using ELISA using plates coated with the respective polysaccharide. The mouse with the highest MenX titer was boosted one more time with the immunogen. Three days later, spleen cells were collected, and after lysis of red blood cells, fusion with SP2/0 myeloma cells at a ratio of 1:1 was performed. In total, 70 million lymphocyte cells were fused with 70 million fusion partner cells and plated on 6 × 96-well plates. These hybridoma cell lines were cultured in 20% RPMI 1640 medium containing hypoxanthine, aminopterin, and thymidine for hybridoma selection. Cell growth was examined 2 weeks after fusion. In the first test, supernatants were screened by ELISA against MenX CPS and 18 positive hybridoma motherwells were further propagated. The hybridoma motherwells were retested the next day, and those with the retained positivity against MenX CPS (7 out of 18) were subsequently expanded and cloned by limiting dilution. Obtained cell lines were cultured and retested for their positivity against 1) MenX-PS, 2) the MenX-PS-CRM_197_ conjugate, 3) the protein carrier, CRM_197_ (GSK, Siena, Italy), 4) an irrelevant polysaccharide antigen, Group B *streptococcus* type II (GSK, Siena, Italy), and 5) another irrelevant polysaccharide antigen, a meningococcal antigen, *Neisseria meningitidis* serogroup A polysaccharide (MenA-CPS) (GSK, Siena, Italy). Only one hybridoma motherwell resulted in the antibody with the desired characteristics. Other attempts to obtain monoclonal antibodies against MenX CPS using eight mice, with minimal variations in the immunzation protocol, did not yield additional antibody clones. Therefore, we generated one monoclonal antibody that specifically recognized MenX-PS and named it clone MenX.01. Large-scale MenX.01 production was performed in RPMI 1640 media (PAN-Biotech GmbH) supplemented with the fetal bovine serum (FBS) standard (PAN-Biotech GmbH) (10%), penicillin–streptomycin (PAN-Biotech GmbH) (final concentrations: penicillin 10 U/mL; streptomycin 10 μg/ml), l-Glutamine (PAN-Biotech GmbH) (final concentration: 0.2 mM), and β-mercaptoethanol 50 mM in PBS (PAN-Biotech GmbH) (final concentration: 5 µM). When they reached confluence, the cells were collected and centrifuged, the medium was replaced with a serum-free medium, and after the next 5–7 days, the antibodies were purified from the supernatant. mAb was purified from the culture supernant using a GE AKTA Pure Liquid Chromatography System and HiTrap Protein G HP prepacked columns for preparative purification of monoclonal antibodies in an amount of a few milligrams.

### Enzymatic Fab Production

Affinity-purified MenX.01 mAb was concentrated to 2 mg/ml in PBS and cleaved into Fab and Fc fragments according to the protocol of Andrew and Titus (Andrew and Titus, 2003). Briefly, the purified antibody stock in PBS (2 mg/ml) was dissolved in a freshly prepared 2x digestion buffer (0.035 M ethylenediaminetetraacetic acid, 40 mM l-cysteine in PBS). Freshly prepared papain (0.1 mg/ml) was mixed in a 1:1 ratio with the antibody in the 2x digestion buffer and incubated (37 C, 2 h). The reaction was stopped by the addition of iodoacetamide to a final concentration of 30 mM. The Fab fragment was purified from papain, the Fc fragment, and the undigested IgG on ÄKTA FPLC *via* tandem Protein G and Protein A affinity purification. Fab fragments were then concentrated in PBS centrifugal Amicon-filter concentrators (molecular weight separation 10 kDa) (Merck KGaA) to a final concentration of 1 mg/ml. The purity of Fab was confirmed by SDS-PAGE analysis, followed by SDS-PAGE and western blot/immunoblot analysis CPS (Supplementary Figure S3A, B).

### Recombinant Fab Production

The construction of the plasmids for recombinant Fab expressions was obtained by sequencing, synthesis, and cloning by GenScript United States Inc. (New Jersey, United States). Briefly, from the selected hybridoma clone, RNA was reverse-transcribed into cDNA. The antibody fragments of variable heavy chains (V_H_) and variable light chains (V_L_) were amplified and cloned into a standard cloning vector separately. Colony polymerase chain reaction was performed to screen for clones with inserts of the correct sizes, and no less than five colonies with the correct insert size were sequenced. The resulting sequence is the consensus derived from the alignment of these clones (S, sequences).

The antibody fragment of V_H_ was synthesized and fused with either the IgG1 or IgG2a first constant heavy chain domain (C_H_1), and the latter was also designed to contain a 6x histidine tag at the C-terminal region. The V_L_ was processed similarly with the IgG constant kappa light chain domain (CL). The synthesized IgG1/IgG2a-kappa heavy and light chains were cloned separately into mammalian expression vector pcDNA3.4.

Transient expression of recombinant Fab was performed in a mono-, tri-, or five-layer cell culture flask (Corning™ Falcon™ Fischer Scientific). HEK293T cells in RPMI 1640 media (PAN-Biotech GmbH) supplemented with the FBS standard (PAN-Biotech GmbH) (10%), MEM NEAA (100x) (PAN-Biotech GmbH), and sodium pyruvate (PAN-Biotech GmbH) (final concentration 0.1 mM) were seeded 24 h before transfection in order to achieve an 80% confluency the next day. For each flask layer, the transfection mixture was prepared by mixing 19 μg of each heavy and light chain purified plasmid, 185 uL of polyethylenimine (PEI) solution (1 mg/ml), and 2.8 ml of Dulbecco’s modified Eagle’s media (PAN-Biotech GmbH) for 20–30 min at RT. Next, the flask media were removed, and the transfection mixture was added; after incubating for 2 min, the media were returned to the culture flask and the flask was placed back in the incubator. After 24 h, the media were exchanged with the HyClone™ HyCell TransFx-H Medium (Cytiva, previously GE Healthcare) supplemented with MEM NEAA (100x) (PAN-Biotech GmbH), sodium pyruvate (PAN-Biotech GmbH) (final concentration: 0.1 mM), penicillin–streptomycin (PAN-Biotech GmbH) (final concentrations: penicillin 10 U/mL; streptomycin 10 μg/ml), and l-Glutamine (PAN-Biotech GmbH) (final concentration: 0.2 mM). Media were collected and replaced every 3–5 days for 1–2 weeks. The recombinant mouse Fabs were purified from the supernatant using a GE AKTA Pure Liquid Chromatography System equipped with a HisTrap HP columns packed with the Ni Sepharose affinity resin. Fab was analyzed by ELISA and western blot to confirm specific binding to MenX CPS (Supplementary Figure S3C, D).

### ELISA

Microtiter plates (96 wells, MICROLON^®^ High Binding, Greiner Bio-One) were coated with polysaccharides (MenX-PS, MenA-PS, GBSII-PS), glycoconjugate MenX-CRM_197_, or the CRM_197_ protein. 100 µL of CPS (5 μg/ml) in PBS pH 8.2 or 50 µL of glycoconjugate/protein (2 μg/ml) in carbonate/bicarbonate coating buffer pH 9.6 was added in each well. Plates were incubated overnight at 2–8 C, washed two times with tap water, and saturated with 150 μL/well PBST-B [3.0% bovine serum albumin (BSA) in PBST (0.05% Tween-20 in PBS pH 7.4)] for 1 h at 37°C. The plates were flicked off to remove the solution and washed twice with tap water. The coated plates were incubated with mAb or Fab thereof in various dilutions at RT for 1 h, washed twice, and incubated for 1 additional hour at RT with either anti-mouse IgG (H + L) Fc peroxidase (Jackson ImmunoResearch) diluted 1:1,000 or anti-mouse IgG F (ab’)_2_ peroxidase (Jackson ImmunoResearch) 1:1,000 diluted in PFT (PBS containing 1% FCS and 0.05% Tween 20) (1% FCS in PBST). After washing six times, the plates were developed with a 0.6 mg/ml solution of *o*-phenylenediamine dihydrochloride (OPD) (Sigma) in citrate buffer pH 5.5 and 0.001% of 30% hydrogen peroxide at RT for 5–10 min. After stopping the reaction with 1M sulfuric acid, the absorbance was measured using a TriStar LB 941 multimode microplate reader with the wavelength set at 490 nm and the reference filter set at 630 nm. ELISA inhibition experiments were performed following the same procedure but preincubating the samples with one or more concentrations of the inhibitor for 20 min at RT.

### Western Blot/Immunoblot Analysis

The CRM_197_ protein or MenX-CRM, MenA-CRM_197_, and GBSII-CRM_197_ glycoconjugates in the amount of 2–10 μg were separated by 8% SDS-PAGE. Fab fragments in the amount of 2–10 μg were separated by 10–12% SDS-PAGE. Samples were transferred onto a 0.45 μm PVDF membrane (Hybond™, GE Healthcare), which were subsequently blocked with 5% w/v blotting grade low-fat powdered milk (Carl Roth Gmbh & Co. Kg). Membranes were incubated with clone MenX.01 (mAb or Fab) overnight at 4 °C. We used our own stock antibodies at a concentration of 1 mg/ml with a typical dilution of the primary antibody being 1:100. Protein signals were developed using anti-mouse IgG F (ab’) 2 peroxidase (Jackson ImmunoResearch) 1:1,000 and visualized with an ImageQuant LAS 4000 mini camera system (GE Healthcare). Fab fragments were developed with either anti-mouse IgG F (ab’)2 peroxidase (Jackson ImmunoResearch) diluted 1:1,000 or anti-mouse IgG (H + L) Fc peroxidase (Jackson ImmunoResearch) diluted 1:1,000 to confirm the absence of the Fc fragment in the preparation.

### Complement-Mediated Bactericidal Activity (Rabbit Serum Bactericidal Activity Assay)

Serum bactericidal activity against *N. meningitidis* serogroup X strain Z9516 was evaluated as reported elsewhere (Giuliani et al., 2005), with minor modifications. Briefly, bacteria were grown overnight on a chocolate agar plate (Biomerieux 43,101) at 37°C in 5% CO_2_. Colonies were inoculated in 7 ml of Mueller–Hinton broth containing 0.25% glucose to an optical density at 600 nm (OD_600_) of 0.05–0.06 and incubated at 37°C with shaking until the early log phase [OD_600_ of ∼0.25 corresponding to 10^9^ colony-forming units per mL (CFU/ml)]. The cultured bacteria were diluted in Dulbecco’s PBS (DPBS- SIGMA D8662) containing 1% bovine serum albumin (BSA) (Sigma) and 0.1% glucose at a working dilution of 10^4^–10^5^ CFU/ml. SBA was run in round-bottom 96-well microplates in a final volume of 50 μL per well with 25 μL of serial twofold dilutions of the test sample (mAb and polyclonal Abs), 12.5 μL of bacteria under the working dilutions, and 12.5 μL of the active complement (25%). The bactericidal assay contains two internal controls: the first is for evaluating the bacterial killing by the complement alone in the absence of antibodies, and the second is for evaluating the killing by serum alone in the presence of a heat-inactivated complement. The reaction mixtures were incubated at 37°C for 60 min with 5% CO_2_; then, each sample was spotted on Mueller–Hinton agar plates. Serum bactericidal titers were defined as the mAb concentrations resulting in a 50% decrease in CFU/mL after a 60 min incubation of bacteria with the reaction mixture compared to the control CFU/mL at time zero.

### Fragments of MenX Polysaccharide Preparation by Mild Hydrolysis

The DP40 OS depolymerization was performed by mild acid hydrolysis. A phosphodiester bond links *N. meningitidis* capsule building blocks, and the hydrolysis of this bond gives rise to a phosphomonoester bond. Therefore, measuring the ratio from the mono- and diester bonds is a way of following the hydrolysis reaction and estimate of the average degree of polymerization (DP) of the sample. The process was monitored by phosphorus (^31^P) NMR spectroscopy, and it was quenched by neutralization when the desired average DP (avDP) was reached. For a MenX OS target of avDP 5, the hydrolysis was performed in 50 mM NaOAc with a saccharide concentration of 2.5 mg/ml at pH 4.0 and 80 C for ∼18 h and two times overnight at RT. The reaction was quenched by neutralization with NaOH when ^31^P NMR indicated an avDP of 11.7.

### Purification of Oligosaccharides

The fragments of different lengths were separated by anionic exchange chromatography using a semipreparative HPLC system with a Sepharose Q column. By increasing the NaCl percentage of the elution buffer with a linear gradient, it was possible to isolate every oligosaccharide fragment in the range of 1–11 repeating units.

The length of the oligosaccharides was determined by ^31^P NMR analysis. The ^31^P NMR signals of phosphodiester in chain groups (P_Int_) and phosphomonoester end groups (P_Ter_) were integrated and used for avDP calculation

$$\text{avDP}=\left[\left(\frac{{\text{P}}_{\text{Int}}}{{\text{P}}_{\text{Ter}}}\right)+1\right]$$

MenX OSs were desalted against water on an SEC Sephadex G-10 column (∼0.3 mg of OS loaded per 1 ml of the resin at 30 cm/h.

### Surface Plasmon Resonance Analysis

Binding kinetics and affinities were determined by SPR using a BIACORE X100 system. Glycoconjugates of MenX were immobilized on research grade CM5 sensor chips (Biacore) using the amine coupling kit supplied by the manufacturer (Biacore). Immobilizations were conducted in 10 mM sodium acetate (pH 5) at a sugar concentration of 30 μg/ml. The immobilized surface density was ∼500 resonance units in each instance. Measurements were conducted in PBS Tween20 0.005% pH = 7.2 at 25 °C and at a flow rate of 45 μL/min. mAb was flown at a 0.3–5.0 µM concentration, while Fab was flown at a 0.6–10 µM concentration. Following mAb or Fab binding, conjugate surfaces were regenerated with 3.5 M MgCl_2_ and a contact time of 120 s. Sensorgram data were analyzed using BIAevaluation software (Biacore). For competitive SPR, PBS Tween 0.005% pH = 7.2 was used as the running buffer for the inhibition assays at a 45 μl/min flow rate at 25°C. The experiment started with three startup cycles to allow surface stabilization. Each sample injection (a 120 s contact time, a 300 s dissociation time) is followed by regeneration with 3.5 M MgCl_2_ (a 120 s contact time) to remove the bound analyte from the ligand immobilized on the chip surface. 10 μM MenX mAb MenX.01 has been used together with descending concentrations of MenX CPS, DP5, and DP2 [500 μg/ml, 250 μg/ml, 125 μg/ml, 62.5 μg/ml, 31.3 μg/ml, 15.6 μg/ml, 7.8 μg/ml, 3.9 μg/ml, 2 μg/ml, 1 μg/ml, 0.5 μg/ml, and 0 μg/ml (no analyte in the mAb solution)].

### Isothermal Titration Calorimetry Measurement

ITC experiments were performed using an ITC200 microcalorimeter (Malvern Panalytical). A volume of 40 μl MenX at different DPs in the syringe was titrated to 200 μl of MenX0.1 mAb in the measurement cell. Each of the MenX fragments DP9, DP7, and DP5.5 was titrated using different molar concentrations of the analyte in a minimum of three experiments and in different buffers (20 mM HEPES at pH 7.4 with 150 mM NaCl, or PBS). Final enthalpograms were collected in PBS, or the same buffer as used in the SPR experiments. Respectively, 150 μM DP5.5 was titrated to 69.1 nM mAb at 292K, 160 μM DP7 to 7.2 μM mAb at 296K, and 167 μM DP9 to 7.2 μM mAb at 296K.

### STD-NMR Experiments

The interactions of mAb MenX0.1 with DP7 in a 1:50 M ratio were studied by STD-NMR using the pulse sequence from the Bruker library (stddiffesgp.3). Spectra were recorded at 600 MHz at RT with 64 scans repeated in 64 loops in a matrix of 32k points in t2 in a spectral window of 6,692.11 Hz centered at 2,820.00 Hz. Excitation sculpting with gradients was employed to suppress the water proton signals. A spin lock filter (T1p) with a 2 kHz field and a length of 30 ms was applied to suppress the protein background. Selective saturation of the protein resonances was performed by irradiating at 7.0 ppm (on-resonance spectrum) using a series of shaped 90° pulses (50 ms, 1 ms delay between pulses) for a total saturation time of 2.0 s. For the reference spectrum (off-resonance spectrum), the irradiation took place at 30 ppm. To obtain the 1D STD-NMR spectra, the on-resonance spectra were subtracted from the off-resonance using Topspin 2.2 software. The difference spectrum corresponds to the STD-NMR spectrum, and the intensity of its signals is proportional to the proximity of the corresponding protons to the protein. STD was analyzed using the amplification factor (*A*_*STD*_). The amplification factor is obtained by multiplying the relative STD effect of a given proton (*I*_*STD*_/*I*_*0*_) at a given ligand concentration ([*L*]_*T*_) with the molar ratio of the ligand in excess relative to the protein ([*L*]_*T*_/[*P*]), according to the equation

$$ASTD=\;\frac{I_{0}-I_{SAT\;}\;}{I_{0}}X\;\frac{{\left[L\right]}_{\text{T}}}{\left[p\right]}=\;\frac{I_{STD\;}}{I_{0}}\;X\;\frac{{\left[L\right]}_{T}}{\left[p\right]}$$

where *A*_*STD*_ is the STD amplification facto and *I*_*0*_, *I*_*SAT*_, and *I*_*STD*_ are the intensities of the reference (off-resonance spectra), saturated (on-resonance spectra), and difference spectra, respectively. In order to get the epitope mapping information from the amplification factor, the relative *A*_*STD*_ with the highest intensity is set for 100%, and all the other signals are normalized accordingly.

### Ab initio Calculations

DFT calculations were carried out with the Gaussian 09 suite of programs. The geometry optimization and the scan analysis were performed utilizing Becke’s hybrid three-parameter exchange functional and the nonlocal correlation functional B3LYP with the 6–31++g (d,p) basis set. Solvent effects were included using the polarizable continuum model (PCM) for water (IEF-PCM). Electronic energies were used to derive the energy profiles around the dihedral angles of interest (φ/ψ and α/β). Scalar coupling constants were computed for all the possible conformations (*exo-syn*, non-*exo*, and *exo*-anti around φ, and gg, gt, and tg around ω) using the GIAO method.

### Molecular Dynamics Simulations

1 µs MD simulations of MenX DP2 and DP12 were performed using the AMBER12 and AMBER16 force fields within GLYCAM06 in explicit water. MenX DP2 and DP12 molecules were built using the GLYCAM carbohydrate builder web tool (http://glycam.org). The phosphate linkers were added using the xleap module of AMBER12, and the parameters and partial atomic charges were calculated with the antechamber module (derived from the DNA phosphodiester bond) using the GAFF force field.

The resulting geometries were extensively minimized using conjugate gradients and then taken as starting structures for the MD simulations in the explicit solvent.

The molecules were solvated in a theoretical box of explicit TIP3P waters, and the solute atoms were positioned at least at 10 Å from the solvent box edge. The equilibration phase consisted of energy minimization of the solvent, followed by an energy minimization of the entire system without restraints. The system was then heated up to 300 K during 100 ps, followed by 2 ns dynamics at a constant temperature of 300 K, controlled using a Langevin thermostat, and a constant pressure of 1 atm. During the simulations, the SHAKE algorithm was turned on and applied to all hydrogen atoms (Miyamoto and Kollman, 1992). A cutoff of 8 Å for all nonbonded interactions was adopted. An integration time step of 2 fs was employed, and periodic boundaries conditions were applied throughout. During the simulations, the particle mesh Ewald (PME) method was used to compute long-range electrostatic interactions (Darden et al., 1993; Essmann et al., 1995; Crowley et al., 1997). Minimization, equilibration, and production phases were analyzed by the pmemd. cuda module of AMBER 12 and 16, while the analyses of the simulations were performed using the cpptraj module from AMBERTOOLS 16 (Götz et al., 2012; Salomon-Ferrer et al., 2013b; Le Grand et al., 2013). Data processing and 2D plots were carried out using GNUplot software.

### Fab Crystallization

Attempts to co-crystallize IgG2a recFab-His with OS DP4 or DP5/6 resulted in crystals under conditions of 0.2 M potassium thiocyanate, 0.1 M Bis-Tris propane, pH 6.5, and 20% PEG 3350 of the PACT premier™ (Molecular Dimensions) screen. The crystals were soaked in the same precipitant solution with an increased PEG 3350 concentration and with added glycerol, 0.2 M potassium thiocyanate, 0.1 M Bis-Tris propane, pH 6.5, 27% PEG 3350, and 10% glycerol and quench-cooled in liquid nitrogen prior to data collection.

X-ray diffraction data were collected at the SOLEIL Synchrotron (Saint-Aubin, France) on beamline PROXIMA 2a using a EIGER X 9M detector. The reflections were indexed and processed using XDS (Kabsch, 2010) and further analyzed using the CCP4 program suite (Winn et al., 2011). The structure of the complex was determined by molecular replacement in Phaser (Mccoy et al., 2007) using template model coordinates from the structure of mouse Fab vFP05.01 (PDB code 5TKK). Refinement and manual model building were performed using Phenix (Liebschner et al., 2019) and COOT (Emsley and Cowtan, 2004), respectively. Data collection and refinement statistics are reported in Supplementary Table S4, Supplemental Information (Williams et al., 2018). Structure quality was assessed using Molprobity and the reflection and coordinate files submitted to the Protein Data Bank (PDB entry code 7OO2). The crystal structure was used for docking the MenX (DP6) hexasaccharide.

### Docking Studies

The MenX (DP6) hexasaccharide was built as already explained for DP2 and DP12. The global minimum conformer obtained from the analysis of DP2 and DP12 was taken as the starting point for the DP6 geometry. The molecule was then solvated in a theoretical box of explicit TIP3P waters; the solute atoms were positioned at least at 10 Å from the solvent box edge, and counterions were added to maintain electroneutrality. The equilibration phase consisted of first an energy minimization of the solvent, followed by an energy minimization of the entire system without restraints, using the steepest descent algorithm. The resulting structure was placed into the CRD of the IgG2a recFab-His crystal structure and manually docked to maximize the intermolecular interactions. The docked structures were then submitted to energy minimization with a low gradient convergence threshold (0.05) in 5,000 steps. The OPLS_2005 force field was employed, as integrated in the MAESTRO (Schroedinger) suite of programs.

All figures were generated using the molecular graphic software PyMOL (The PyMOL Molecular Graphics System, Version 2.4 Schrödinger, LLC, http://www.pymol.org).

### *In vivo* Studies

Protocols were approved by the Italian Ministry of Health (Approval number n. 804/2015-PR). All mice were housed under specific pathogen-free conditions at the GSK Vaccines Animal Resource Center in compliance with the relevant guidelines (Italian Legislative Decree n 26/2014). Three groups of eight female BALB/c mice (Charles River) were immunized by subcutaneous injection of glycoconjugates at a 1 µg dose in saccharide content using alum phosphate as an adjuvant. The adjuvant AlPO_4_ (manufactured in GSK) was administered at 0.12 mg/dose. Mice received the vaccines at days 1, 14, and 28. Mice were bled at days 0, 27, and 42.

## Data Availability

The datasets presented in this study can be found in online repositories. The name of the repository and accession number can be found below: The Research Collaboratory for Structural Bioinformatics (RCSB) Protein Data Bank (PDB), https://www.rcsb.org/, 7OO2
