# Supplementary material for: Elucidating the Structural and Minimal Protective Epitope of the Serogroup X Meningococcal Capsular Polysaccharide
Source: Front Mol Biosci. 2021 Oct 14;8:745360. doi: 10.3389/fmolb.2021.745360 (PMC8551719; doi:10.3389/fmolb.2021.745360)
Supplement: Supplementary file 1 [file DataSheet1.PDF]

Supplemental Information

Elucidating structural and minimal protective epitope of serogroup X meningococcal capsular polysaccharide

Gian Pietro Pietri[a], Marta Tontini[b], Barbara Brogioni[b], Davide Oldrini[b], Stefania Robakiewicz[c], Pedro Henriques[b], Ilaria Calloni[d], Vera Abramova[a], Laura Santini[b], Suzana Malić[a], Karmela Miklić[a], Berislav Lisnic[a], Sara Bertuzzi[d], Luca Unione[d], Evita Balducci[b], Jérôme de Ruyck[c], Maria Rosaria Romano[b], Jesus Jimenez Barbero[d], Julie Bouckaert[c], Tihana Lenac[a], Stipan Jonjic[a*], Roberto Adamo[b*]

[a]*Center for Proteomics, Faculty of Medicine, University of Rijeka, Rijeka, Croatia*

[b]*GSK Vaccines, Via Fiorentina 1, 53100 Siena, Italy*

[c]*Unité de Glycobiologie Structurale et Fonctionnelle, UMR 8576 du CNRS et Université de Lille, 50 Avenue de Halley, 59658 Villeneuve d'Ascq, France*

[d]*Chemical Glycobiology Lab CIC bioGUNE Technology Park, 48160 Derio, Spain*

[e]*Ikerbasque, Basque Foundation for Science, 48013 Bilbao, Bizkaia, Spain*

[f]*Department of Organic Chemistry II, University of the Basque Country, Universidad del País Vasco/Euskal Herriko Unibertsitatea, 48940 Leioa, Bizkaia, Spain*

[*] Corresponding authors e-mail: tihana.lenac@uniri.hr; roberto.x.adamo@gsk.com.

Sequence of the recombinant murine MenX.01 Fabs	Pg S2
Serum Bactericidal activity of the mAb	Pg S3
Conformational studies on MenX CPS	Pg S3
Characterization of MenX oligosaccharides	Pg S4
Fab generation	Pg S5
Isothermal titration calorimetry and Surface Plasmon Resonance	Pg S5
Crystal structure of the anti-MenX Fab	Pg S7
Docking on the crystal structure of the anti-MenX Fab	Pg S10
Characterization of MenX glycoconjugates	Pg S11
References	Pg S11

Sequence of the recombinant murine MenX.01 Fabs

Heavy chain with murine IgG1 CH_1 constant region:

MGWSCIILFL VATATGVHS**Q VQLKESGPGL VAPSQSLSIT CTVSGFSLSR** 50
YSVHWVRQPP GKGLEWLGMI WGGGSTDYNS ALKSRLSISK DNSKSQVFLK 100
MNSLQTDDTA MYYCARNYRG FAYWGQGTLV TVSAAKTTPP SVYPLAPGSA 150
AQTNSMVTLG CLVKGYFPEP VTVTWNSGSL SSGVHTFPAV LQSDLYTLSS 200
SVTVPSSTWP SETVTCNVAH PASSTKVDKK IVPRDCGK 238

The signal peptide is underlined; **the variable sequence is shown in bold**.

Heavy chain with murine IgG2a CH_1 constant region:

MGWSCIILFL VATATGVHS**Q VQLKESGPGL VAPSQSLSIT CTVSGFSLSR** 50
YSVHWVRQPP GKGLEWLGMI WGGGSTDYNS ALKSRLSISK DNSKSQVFLK 100
MNSLQTDDTA MYYCARNYRG FAYWGQGTLV TVSAAKTTAP SVYPLAPVCG 150
DTTGSSVTLG CLVKGYFPEP VTLTWNSGSL SSGVHTFPAV LQSDLYTLSS 200
SVTVTSSTWP SQSITCNVAH PASSTKVDKK IEPRGPTIKP 240

The signal peptide is underlined; **the variable sequence is shown in bold**.

Light chain with murine kappa constant region:

MGWSCIILFL VATATGVHS**E TTVTQSPASL SVATGEKVTI RCITSTDIDD** 50
DMTWYQQKPG EPPKLLISEA TTLRPGVPSR FSASGYGTDF VFTIENTLSE 100
DVADYYCLQS DNMPYTFGGG TKLEIKRADA APTVSIFPPS SEQLTSGGAS 150
VVCFLNNFYP KDINVKWKID GSERQNGVLN SWTDQDSKDS TYSMSSTLTL 200
TKDEYERHNS YTCEATHKTS TSPIVKSFNR NEC 233

The signal peptide is underlined; **the variable sequence is shown in bold**.

Serum Bactericidal activity of the mAb

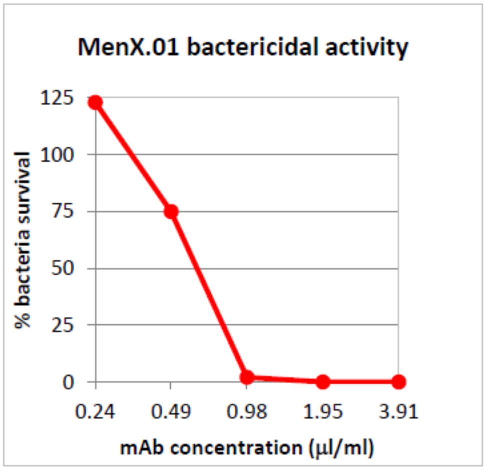

Figure 1. Determination of the Serum Bactericidal Titers of the mAb MenX0.1 as perrcentage of bacterial survival at different mAb concentrations.

Conformational studies on MenX CPS

Table S1. Combinations of φ, ψ, α and β for the global minimum (1) and the local minima (2-4) of DP2.

Structure	ϕ	ψ	α	β
1	60	60	-50	60
2	60	60	-50	-60
3	60	-60°	0	180
4	60	-60°	-50	60

The theoretical values were calculated through the GIAO method at the B3LYP/6-31++g(d,p) level. The experimental values agree with an equilibrium between the *exo-syn* (> 80%) and the *exo-anti* (< 20%) conformations.

	$^{3}J_{H1-P'}$	$^{3}J_{P-C2}$	$^{2}J_{P-C1}$
Experimental	6	7,9	5,9
DFT calculation for *exo-syn*	4,3	7,4	2,1
DFT calculation for *non-exo*	0,3	0,2	7,2
DFT calculation for *exo-anti*	18	0,1	7

Table S2. Theoretical and experimental J couplings (in Hz) for the staggered orientations around □/□ of the DP2 disaccharide**.** The theoretical values were calculated through the GIAO method at the B3LYP/6-31++g(d,p) level. The experimental values agree with an equilibrium among different conformations with a major population of the the a=0/b=-60 conformation is the major one.

	${}^{2}J_{P\text{-}C4}$	${}^{3}J_{P\text{-}H4}$
Experimental	5,4	9.2
DFT calculation for α=0 and β=-60	3,6	8,5
DFT calculation for α=180 β=60	6,2	19
DFT calculation for α=-25 β=-60	4,2	6,8
DFT calculation for α=-25 β=60	5,7	7,5

Characterization of MenX oligosaccharides

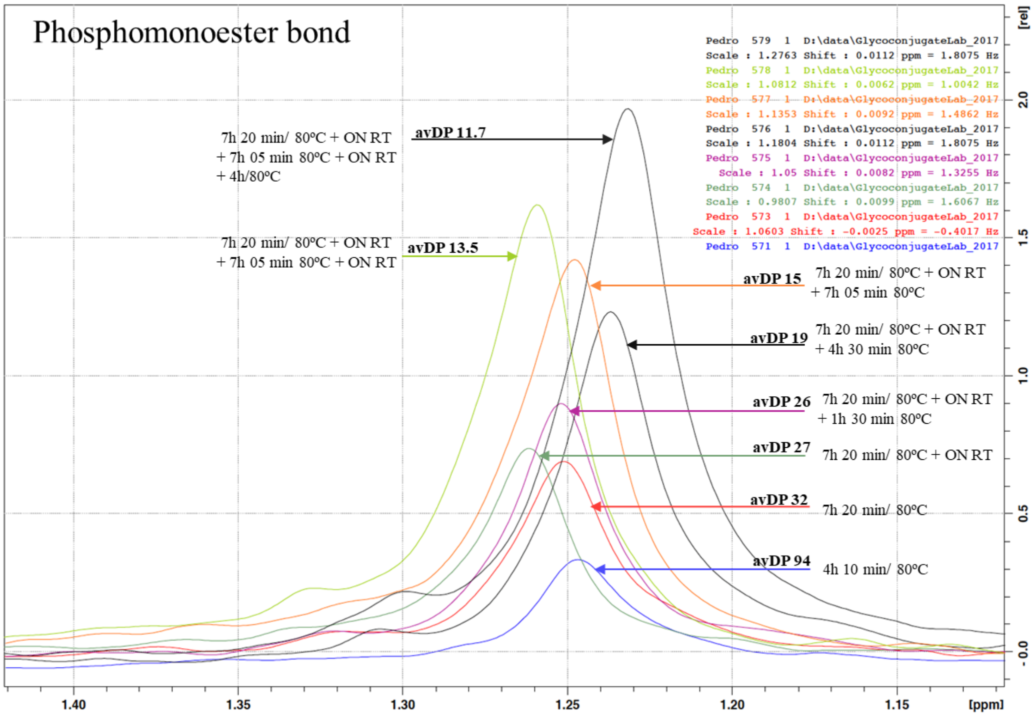

Figure S1. ${}^{31}P$ NMR (D_2O, 400 MHz) monitoring of acid hydrolysis of MenX CPX at different time points obtain different size OS obtained.

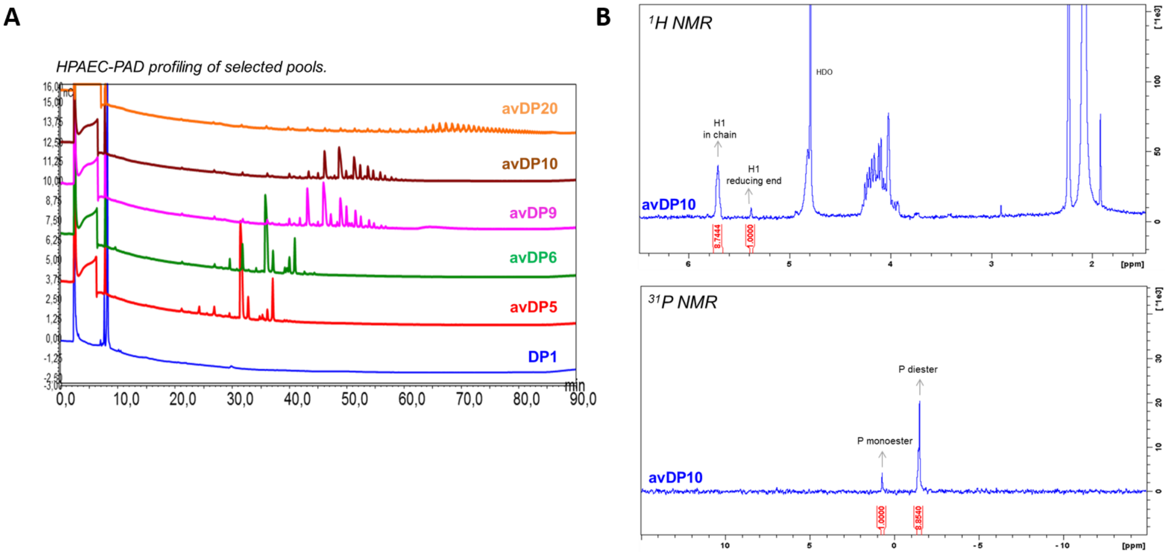

Figure S2. Characterization of MenX OS obtained from CPS MenX acid hydrolysis: A) HPAEC-PAD prifiling to determine average degree of polymerization. B) ^{1}H and ^{31}P NMR to determine the DP according to the formula The DP was calculated as $[(P_{Intraglycosidic}/P_{Terminal}) + 1]$.

Fab generation

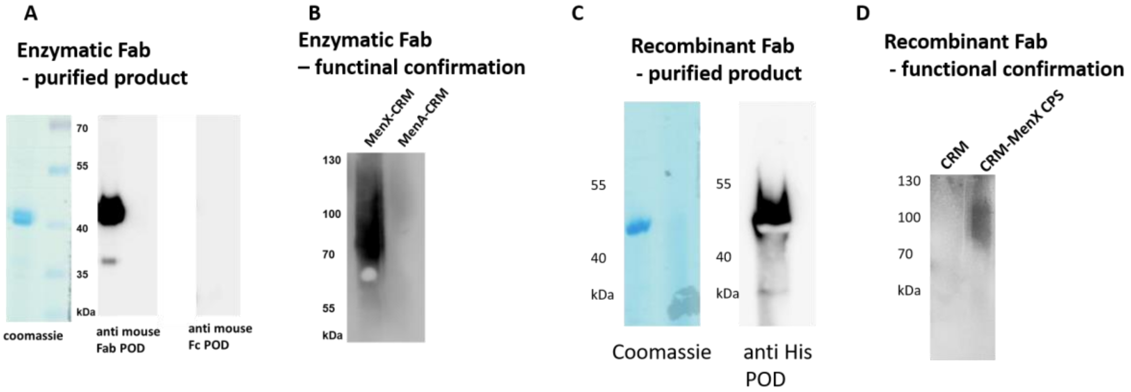

Figure S3. SDS Page and western blot of the Fabs obtained by different methodologies.

Isothermal titration calorimetry and Surface Plasmon Resonance

Measurements of the enthalpy of the binding of the MenX polysaccharideto the monoclonal antibody anti-MenX0.1 were performed using a ITC200 microcalorimeter (Malvern Panalytical). A volume of 40 µl MenX, at different degrees of polymerization (DP), in the syringe was titrated to 200 µl MenX0.1 mAb in the measurement cell, as

follows: 150 µM DP5/6 to 69.1 nM mAb, 160 µM DP7 to 7.2 µM mAb and 167 µM DP9 to 7.2 µM mAb.

Table S3. Thermodynamic parameters of differently sized OS binding the mAb, compared with the SPR data

**SPR**	**K_d (µM)**	**k_a (10^3 M-1s-1)**	**k_d (10^{-3} s-1)**	**R_{max} (RU)**	**Chi^2**	**U-value**
avDP15 (mAb)	0.32 ± 0.04	8.64 ± 0.32	2.75 ± 0.06	9.92 ± 0.17	3.08	12
avDP15 (FAb)	0.54 ± 0.04	4.99 ± 0.15	2.71 ± 0.05	9.12 ± 0.13	1.95	9
**ITC (mAb)**	**K_d (µM)**	**Δ H (cal/mol)**	**TΔS (cal/mol)**	**Δ G (cal/mol)**	***n* (molar ratio)**	
DP9	0.80 ± 1.33	-1327 ± 455.1	6980	-8235	1.95 ± 0.394	
DP7	3.82 ± 6.69	-1420 ± 911.0	5904	-7324	2.05 ± 0.874	
DP5.5	2.35 ± 3.36	-1039 ± 318.2	6464	-7504	2 (n fixed for fit)	

$\Delta G = \Delta H - T\Delta S$, $\Delta G = RT\ln(K_d)$

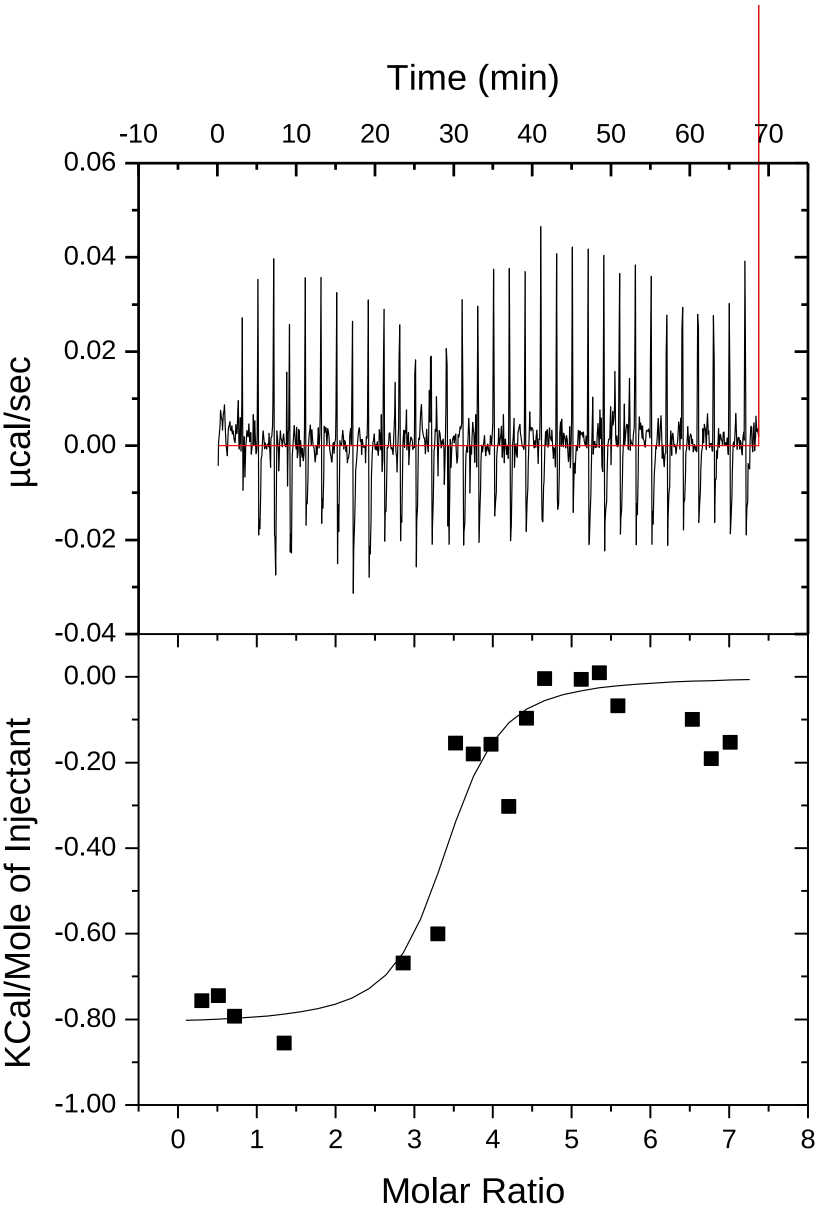

Figure S4. Isothermal titration of MenX DP7 at 160 µM to MenX.01 mAb at 7.2 µM, in PBS buffer, at 22.05372 degrees Celsius.

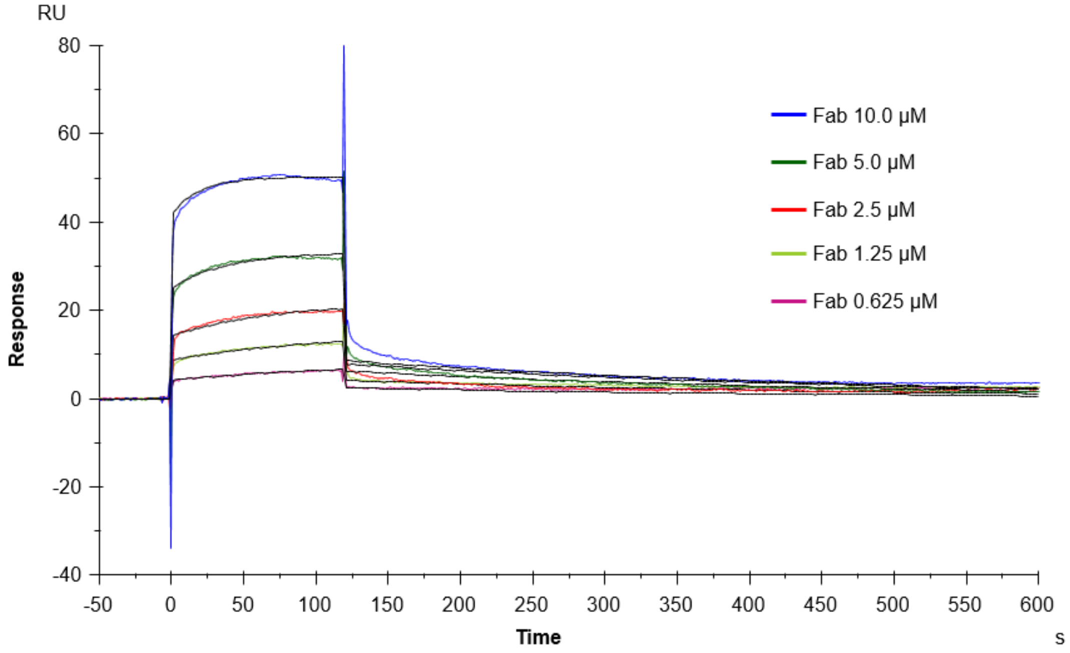

Figure S5. SPR sensorgrams of the interactions between the Fab and MenX avDP15, conjugated to CRM_{197} and immobilized on a CM5 chip via EDC-NHS coupling chemistry (at pH 5) at a level of 458 RUs. The Fab qwas flown at 0.6-10 µM concentration. PBS with 0.005% Tween at pH=7.2 was used as the running buffer. Contact time = 120s, Dissociation time = 300s. Rmax = 9.12 ± 0.13 RU.

Crystal structure of the anti-MenX Fab

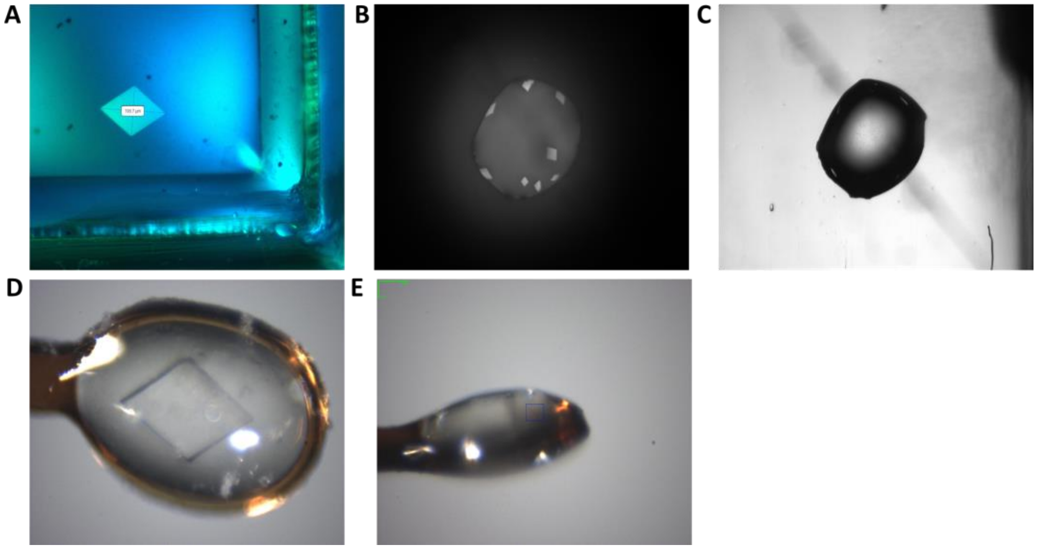

Figure S6 (A-E). Phases of generation of crystals from MenX Fab[1,2].

Table S4. Data collection and refinement statistics (PDB entry code 7OO2)[a].

	Anti-MenX Fab
**Wavelength (Å)**	0.9786
**Resolution range (Å)**	49.49 - 2.16 (2.237 - 2.16)
**Space group**	P 21 21 21
**Unit cell (Å)**<br>**(°)**	83.01 85.35 123.27<br>90 90 90
**Total reflections**	654752 (66304)
**Unique reflections**	47689 (4692)
**Multiplicity**	13.7 (14.1)
**Completeness (%)**	99.93 (99.10)
**Mean I/sigma(I)**	23.09 (1.99)
**Wilson B-factor**	55.41
**R-merge**	0.063 (1.310)
**R-meas**	0.066 (1.219)
**CC1/2**	0.988 (0.708)
**Reflections used in refinement**	47665 (4694)
**Reflections used for R-free**	1992 (197)
**R-work**	0.2132 (0.3748)
**R-free**	0.2620 (0.3926)
**CC(work)**	0.961 (0.649)
**CC(free)**	0.940 (0.626)
**Number of non-hydrogen atoms**	7008
**macromolecules**	6588
**ligands**	9

**solvent**	411
**Protein residues**	864
**RMS(bonds)**	0.004
**RMS(angles)**	0.72
**Ramachandran favored (%)**	95.79
**Ramachandran allowed (%)**	4.09
**Ramachandran outliers (%)**	0.12
**Rotamer outliers (%)**	2.24
**Clashscore**	3.47
**Average B-factor**	64.37
**macromolecules**	64.28
**ligands**	86.59
**solvent**	65.33

a. Statistics for the highest-resolution shell are shown in parentheses.

Docking on the crystal structure of the anti-MenX Fab

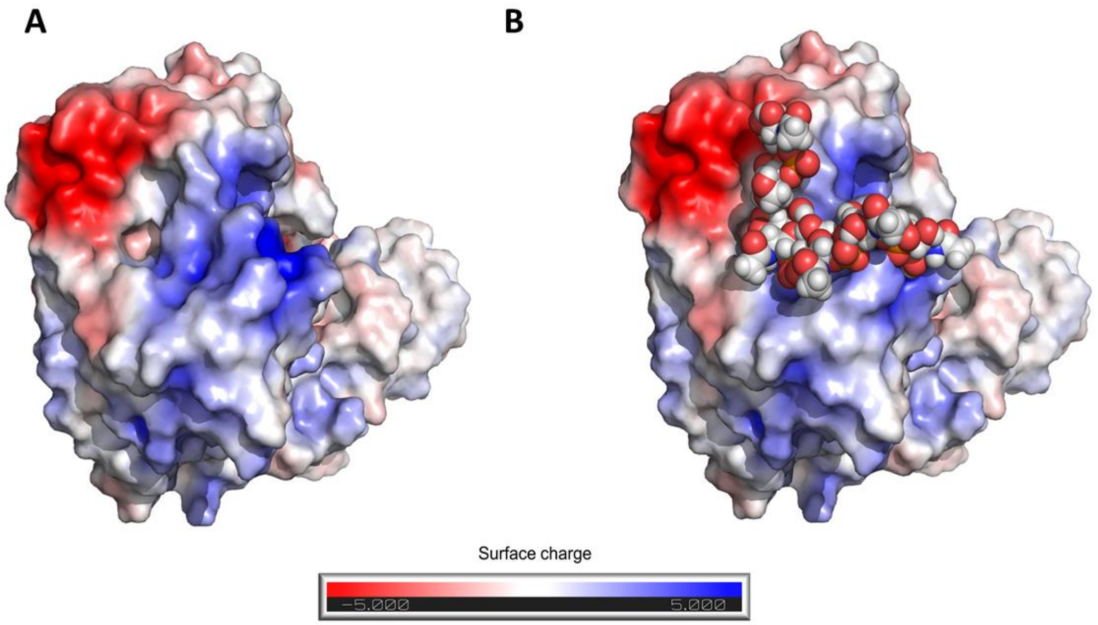

Figure S7. Electrostatic surface of the MenX.01 Fab in its (A) apo and holo (B) forms. The negatively charge phosphodiester groups of the DP6 largely complement the positively charged surface at the CRD.

Characterization of MenX glycoconjugates

A

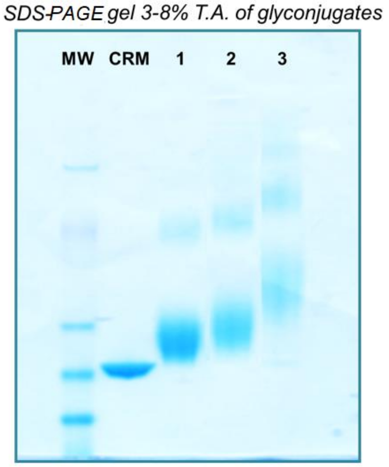

B

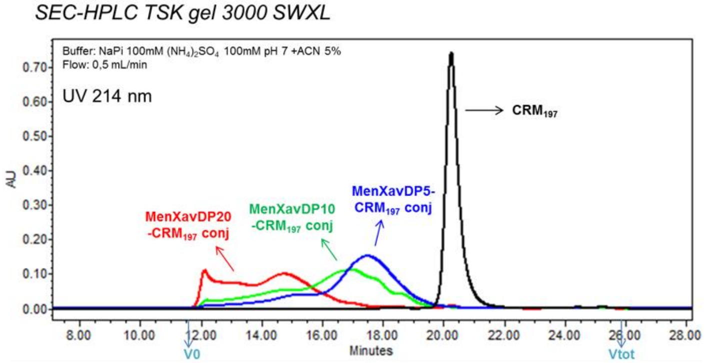

C

Entry	Sample	Sacc/Prot mol/mol	Sacc/Prot w/w
1	MenXavDP5-CRM$_{197}$	3.6	0,093
2	MenXavDP10-CRM$_{197}$	2.9	0,12
3	MenXavDP20-CRM$_{197}$	2.7	0,27

Figure S8. Characterization of the synthesized MenX glycoconjugates. A) SDS-PAGE of the obtained conjugates using CRM197 as control; T.A. is tris acetate. B) SEC-HPLC profiles of the prepared conjugates in comparison to the unconjugated protein. C) Table summarizing the conjugates characteristics.

SEC-HPLC method for glycoconjugates analysis

SEC-HPLC analysis was performed on a Ultimate 3000 system (Dionex-Themo Fisher) equipped with IR and UV 214, 280, 254 nm detectors and with a Tosoh TSKgel G3000SWXL 7.8 mm ID x 30 cm L column + guard. An isocratic elution was made with buffer 100 mM NaPi 100 mM Na_2SO_4 pH 7.2 + 5% ACN (MP) at 0.5 mL/min. Samples were analyzed at a protein concentration of 0,3 mg/mL in mobile phase, the injection volume was 50 µL and the length of each run was 50 minutes. Chromatographic data were elaborated by using Chromeleon™ software vs7.2.

References

1. . Kunik, S. Ashkenazi, and Y. Ofran, "Paratome: an online tool for systematic identification of antigen-binding regions in antibodies based on sequence or structure," Nucleic Acids Res, vol. 40, no. Web Server issue, pp. W521-4, Jul 2012, doi: 10.1093/nar/gks480.

2. O. Haji-Ghassemi, R. J. Blackler, N. Martin Young, and S. V. Evans, "Antibody recognition of carbohydrate epitopes," Glycobiology, vol. 25, no. 9, pp. 920-52, Sep 2015, doi: 10.1093/glycob/cwv037.
